# Origin and cross-century dynamics of an avian hybrid zone

**DOI:** 10.1186/s12862-017-1096-7

**Published:** 2017-12-15

**Authors:** Andrea Morales-Rozo, Elkin A. Tenorio, Matthew D. Carling, Carlos Daniel Cadena

**Affiliations:** 1 Laboratorio de Biología Evolutiva de Vertebrados, Departamento de Ciencias Biológicas, Universidad de Los Andes, Bogotá, Colombia; 2 Calima: Fundación para la Investigación de la Biodiversidad y Conservación en el Trópico, Cali, Colombia; 3 Cornell Laboratory of Ornithology, Cornell University, Ithaca, NY USA; 4 Department of Zoology and Physiology, University of Wyoming, Laramie, WY USA; 5 Programa de Biología y Museo de Historia Natural, Universidad de los Llanos, Sede Barcelona, Villavicencio, Colombia

**Keywords:** Andes, Cline, Hill function, Distribution modeling, Hybridization, Moving hybrid zone

## Abstract

**Background:**

Characterizations of the dynamics of hybrid zones in space and time can give insights about traits and processes important in population divergence and speciation. We characterized a hybrid zone between tanagers in the genus *Ramphocelus* (Aves, Thraupidae) located in southwestern Colombia. We evaluated whether this hybrid zone originated as a result of secondary contact or of primary differentiation, and described its dynamics across time using spatial analyses of molecular, morphological, and coloration data in combination with paleodistribution modeling.

**Results:**

Models of potential historical distributions based on climatic data and genetic signatures of demographic expansion suggested that the hybrid zone likely originated following secondary contact between populations that expanded their ranges out of isolated areas in the Quaternary. Concordant patterns of variation in phenotypic characters across the hybrid zone and its narrow extent are suggestive of a tension zone, maintained by a balance between dispersal and selection against hybrids. Estimates of phenotypic cline parameters obtained using specimens collected over nearly a century revealed that, in recent decades, the zone appears to have moved to the east and to higher elevations, and may have become narrower. Genetic variation was not clearly structured along the hybrid zone, but comparisons between historical and contemporary specimens suggested that temporal changes in its genetic makeup may also have occurred.

**Conclusions:**

Our data suggest that the hybrid zone likey resulted from secondary contact between populations. The observed changes in the hybrid zone may be a result of sexual selection, asymmetric gene flow, or environmental change.

**Electronic supplementary material:**

The online version of this article (doi: 10.1186/s12862-017-1096-7) contains supplementary material, which is available to authorized users.

## Background

Characterizations of hybrid zones allow one to make inferences about traits and processes relevant to understanding the origin and maintenance of differences between populations and species [1, 2]. A classic question about hybrid zones is how are they formed, with previous studies proposing two main hypotheses (reviewed by [3]). The hypothesis of secondary contact posits that hybrid zones result from expansion of populations that were previously isolated geographically and which interbreed in contact zones because complete reproductive isolation between them was not reached during the allopatric phase [4]. An alternative hypothesis postulates that hybrid zones form in parapatry, by primary differentiation across ecological gradients [5]. Secondary contact is likely if environments that presently allow the distributions of hybridizing populations to overlap were disjunct in the past, a scenario that predicts one should observe genetic signatures of demographic expansions. Alternatively, primary differentiation along a gradient would occur if the extent of suitable environments for the hybridizing populations has been stable over time; this predicts that populations have not expanded their ranges historically, and that the position of the hybrid zone (as indicated by the position of clines in molecular and morphological traits) is coincident with an environmental transition [1, 6].

Inferring hybrid-zone origins from current patterns of variation is challenging because, with time, genetic signatures of secondary contact or primary intergradation tend to erode [3, 7]. Alternatively, then, tests of hypotheses posed to account for the origin of hybrid zones may be conducted by examining the historical distribution of hybridizing taxa using paleodistributional modeling, an approach employing niche models that characterize the current distribution of species in climatic space to infer historical potential distributions given climatic conditions of the past [8, 9]. Such models of historical distributions represent hypotheses one can further test using molecular data to evaluate their population-genetic predictions, such as signatures of population growth for presumably expanding populations and of constant population size and isolation by distance in populations occurring within climatically stable areas [10, 11]. This approach has revealed that several hybrid zones likely originated following range expansions leading to secondary contact [12–15].

Another focus of studies on hybrid zones is the analysis of their temporal dynamics, which can allow understanding the role played by different evolutionary forces in such scenarios. When hybrid genotypes are less fit than parental genotypes, ‘tension zones’ are formed, which are maintained by a balance between the homogenizing effect of dispersal into the hybrid zone and the diversifying effect of selection against hybrids [1]. If there is endogenous selection against hybrids, then there should be coincidence in location and concordance in width of clines describing the variation in different traits and loci across a hybrid zone, and such clines should remain stable over time [16, 17]. However, hybrid zones are often temporally dynamic (i.e. they may shift in location or change in width) and because clines for different traits may change in different ways, one can make inferences about the action of particular processes (e.g., natural selection, sexual selection, competition, asymmetric hybridization, dominance drive) based on dynamics observed for different characters [18]. For example, discordant patterns of plumage and mitochondrial DNA variation across a hybrid zone between *Setophaga* warblers, coupled with behavioral experiments showing aggressive superiority of males of one species over the other, indicate that movement of this zone has likely been driven by competition-mediated asymmetric hybridization [19–21]. Temporal changes in the makeup of hybrid zones may also reflect natural or human-mediated environmental changes [22, 23].

**Fig. 1:**
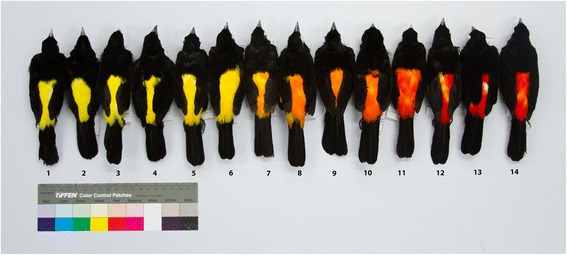
Phenotypic variation in male specimens collected along the *Ramphocelus flammigerus* hybrid zone in southwestern Colombia. Individuals 1-6 correspond to *R. flammigerus icteronotus* (yellow-rumped form) from the plains of the Pacific coast (sector 1; see Fig. 2). On the other extreme, individuals 11-14 correspond to *R. flammigerus flammigerus* (scarlet-rumped form) distributed towards the Cauca River Valley (sector 3). Individuals 7-10 are intermediates collected near the center of the hybrid zone (sector 2)

**Fig. 2:**
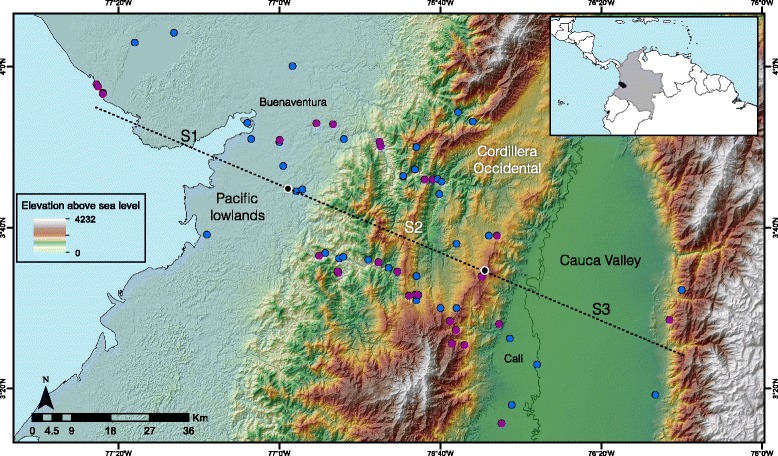
Study transect encompassing the *Ramphocelus* hybrid zone from the Pacific lowlands to the eastern slope of the Cordillera Occidental of the Colombian Andes. Blue dots indicate collection sites of historical specimens and purple dots indicate collection sites of current specimens sampled for phenotypic/genetic variation. The extent of each of the three sectors we defined in the hybrid zone (S1, S2 and S3; see text) is indicated by black dots on the transect

There is ample evidence of hybridization between members of the tanager genus *Ramphocelus* (Aves, Thraupidae; [24–27]), but detailed studies on hybrid zones involving species in this group are scant. Here, we characterize a hybrid zone between members of this genus located in western Colombia that has received little study although its existence was noted nearly a century ago [28] and was described in some detail more than five decades ago [25]. In the Cauca River Valley above c. 900 m elevation, one finds the larger form *flammigerus* (males are black with a scarlet rump), whereas the smaller and yellow-rumped form *icteronotus* occurs along the costal plains west of the Andes extending north into Costa Rica and south into northern Peru. Females and immatures are similar to their respective males, but are less strongly colored. Along a c. 140 km transect running approximately northwest from the city of Cali downslope along the western flank of the Cordillera Occidental, the two forms hybridize, forming a gradient in coloration and body mass ([25, 29]; Figs. 1 and 2). Currently, these forms are considered subspecies of *Ramphocelus flammigerus* [30] because gene exchange between them along this transect appears to be unrestricted, with seemingly no selection against hybrids [25]. We have observed that the two forms also meet and hybridize in contact zones located in low passes further north in the Cordillera Occidental (i.e., Risaralda and Antioquia departments), but such zones have not been studied in detail. Both taxa favor overgrown pastures and shrubby forest edges yet differ ecologically in their elevational distributions, with *flammigerus* ranging from 800 to 2000 m and *icteronotus* from sea level mostly to 1400 m, with occassional records up to 2100 m [31].

The *R. flammigerus* system is particularly well suited to studying the role of different evolutionary forces at work in hybrid zones owing to the existence of variation in characters with different modes of inheritance, and, presumably, under different forms of selection. Variation in rump coloration across the hybrid zone likely reflects variation in the concentration of a single carotenoid pigment and is influenced by the environment because carotenoids are obtained from the diet [25, 32, 33]. Furthermore, based on the strong sexual dichromatism in this species, and the likelihood that its mating system involves some degree of polygamy [34], plumage coloration is probably influenced by sexual selection. In contrast, morphometric variation [25, 29] likely has a strong genetic basis [35–37] and could be subject to natural selection [38–40]. The value of considering traits or loci with different modes of inheritance and under different selective pressures to understand evolutionary forces at work in hybrid zones is illustrated by studies showing (1) that clines for traits involved in courtship are displaced with respect to clines for presumably neutral traits or loci, suggesting a role for sexual selection driving introgression [41, 42]; (2) that sex-linked molecular markers introgress over shorter distances than autosomal markers, suggesting a role for sex chromosomes in reproductive isolation [43, 44]; or (3) that there is more limited introgression in organellar DNA than in nuclear genes, suggesting selection acts more strongly on hybrids of the heterogametic sex (Haldane’s rule; [45]).

Here, we sought to evaluate whether the *Ramphocelus* hybrid zone in southwestern Colombia originated as a result of secondary contact or of primary differentiation, and to examine the zone’s dynamics over nearly a century to make inferences about the action of different evolutionary processes. To accomplish this, we (1) reconstructed the biogeographic and demographic history of the hybridizing populations based on ecological niche modeling and coalescent analyses of mtDNA sequence data, (2) characterized genetic, morphological and plumage variation across the hybrid zone, and (3) compared spatial patterns of variation in morphometrics, plumage coloration, and genetic structure between specimens collected at different times to assess possible changes in the position and width of the hybrid zone.

## Methods

### Samples

We characterized the *Ramphocelus* hybrid zone historically by examining specimens collected from 1894 to 1986 in the ornithological collections of the American Museum of Natural History, the Cornell University Museum of Vertebrates, Universidad del Valle, and the Instituto de Ciencias Naturales at Universidad Nacional de Colombia. To describe current patterns of variation, we collected 73 new specimens in 2007-2010: 65 of them are from localities ranging across the hybrid zone over a distance of 140 km by road connecting the cities of Cali and Buenaventura in department Valle del Cauca [25]; the remaining eight are from localities distant from the hybrid zone in the departments of Antioquia (3), Risaralda (3), and Cauca (2). Study skins and tissue samples are deposited in the Museo de Historia Natural de la Universidad de los Andes (ANDES, Additional file 1: Table S1). All specimen localities (historical and current) were plotted and their position along a transect line that best adjusted to points (estimated using a linear regression between latitude and longitude) was recorded. To construct character clines, we recorded the perpendicular position of each specimen on the regression line (Fig. 2; Additional file 1: Table S1) and calculated the distance from the northwest extreme of our study transect on the Pacific coast to the position of each specimen along the line.

### Biogeographic history

To model potential distributions of the study taxa, we used 343 georeferenced localities obtained from museum specimens and reliable field observations from Costa Rica, Panama, Colombia, Ecuador, and Peru ([46]; GBIF Data Portal, C. Sánchez, pers. comm., our observations). We associated localities with GIS layers for 19 climate variables at a c. 1 km resolution developed for the present (WorldClim; [47]). With these data, we used a maximum entropy approach (Maxent; [48]) based on current climate layers to generate models of the ecological niche and potential distribution of *flammigerus* and *icteronotus* at present. We conducted analyses in which localities of both forms and presumed hybrids were considered together to build potential distribution models, and also built separate models using data for *flammigerus*-like and *icteronotus-*like specimens; intermediate individuals were excluded from the latter analyses. Model performance in predicting present distributions (evaluated using receiver-operating-characteristic curves; [49]) was satisfactory (see below), validating the use of this approach to infer potential distributions in the past.

We projected models based on current climate data onto historical climate surfaces for 6000 years ago and for the Last Glacial Maximum (LGM; 21,000 years ago) to determine whether the distributions of our study taxa were likely disjunct in the past as predicted by the secondary contact hypothesis, or have likely been continuous as predicted by the primary intergradation hypothesis. This approach requires assuming ecological niche conservatism and that climate represents a long-term stable constraint on potential distributions. Because ecological niche models are based only on climatic data, they tend to overpredict potential distributions into areas where the study species do not occur owing to historical limitations to dispersal (e.g., the Amazon region in *R. flammigerus*). To reduce overprediction, we cropped maps of current and historical distributions to include only areas within the Andes Ecoregion [50]. Although cropping potential distributions to this region probably did not remove all areas of model overprediction, it allowed for a semi-quantitative comparison of potential distributions across different time periods by calculating the extent of presence areas within the ecoregion. Maxent produces a continuous output ranging from zero to one describing the probability of the species potentially being present at different sites. We considered a threshold of 10% omission to categorize pixels as suitable or unsuitable for each of the time periods.

### Genetic characterization and demographic history

We analyzed variation in DNA sequences of the cytochrome *b* mitochondrial gene for 58 of the *flammigerus/icteronotus* individuals collected in Colombia from 2007 to 2010. In addition, we obtained sequences for three individuals from Ecuador and two from Panama, and combined our data with two sequences of *flammigerus* available in GenBank: one from Ecuador (accession U15719.1; [51]) and one from Panamá (FJ799882.1; [52]; Additional file 1: Table S1).

DNA was extracted from tissue samples or toepad samples taken from specimens using a Qiagen DNeasy Tissue Kit or a phenol-chloroform method [53]. PCRs used primers H16064 and L14996 [54] in 24 μl amplification reactions using the following conditions: 42 ng of DNA, 0.416 mM dNTPs, 0.5 mM of each primer, 1.042 units of 10X buffer with 1.56 mM MgCl2, 0.0246 units/ml AmpliTaq DNA polymerase), and 16.5 of sterile ddH2O. Reactions began with an initial denaturation at 94 °C for 2 min, followed by 34 cycles of denaturation at 94 °C for 30 s, annealing at 52 °C for 30 s, and extension at 72 °C for 1 min, with a final extension phase at 72 °C for 7 min. PCR products were purified with Affymetrix Exosap-IT and sequenced in both directions. Sequences were edited, assembled and aligned using Geneious Pro 3.6.1 (http://www.geneious.com). The mean length of these sequences was 988.8 bp (range 888-1008 bp).

We also analyzed mtDNA from historical toepad samples of 87 specimens collected by C. G. Sibley in 1956 and housed at the Cornell University Museum of Vertebrates [25]. DNA extraction and amplification of these samples was carried out in a historical DNA lab, following protocols to reduce the odds of contamination [55]. For these specimens, we amplified and sequenced c. 210 bp of the cytochrome *b* gene (mean = 209.9, range = 203-210 bp).

We examined genealogical relationships among haplotypes observed in *flammigerus* and *icteronotus* at present using a maximum-likelihood (ML) phylogenetic analysis employing the GAMMA model. Nodal support was estimated using 1000 bootstrap replicates in RAxML, run from the RAxML BlackBox Web-Server [56]. We used as outgroups sequences of *Ramphocelus carbo* (AF310048.1; [57]) and *R. passerinii* (EF529965.1; [52]).

To assess potential changes in the genetic makeup of the hybrid zone over time, we examined population structure separately for the 1956 specimens and for our samples collected in 2007-2010 (hereafter 2010 specimens) employing procedures implemented in the program ARLEQUIN v3.5 [58]. Based on each data set, we conducted analyses of molecular variance (AMOVAs). We divided our sampling transect in three sectors of equal length: sector 1, 0 – 44 km; sector 2, 45-89 km; and sector 3, 90-134 km (Fig. 2). Within each sector, we grouped individuals collected within 1 km from each other in a single locality. We calculated F-statistics to estimate differentiation among sectors (F_CT_), among localities within sectors (F_SC_), and among localities among sectors (F_ST_). Given that we lacked extensive sampling within point localities that would allow us to examine changes in genetic structure at a fine scale, this analysis allowed us to examine whether there has been any change in the way in which genetic variation is grossly distributed within and among different parts of the hybrid zone; if spatial genetic structure has become eroded (e.g., if introgressive hybridization has led to genetic homogenization across the transect), then one would expect an increase in genetic variation existing within sectors and a decrease in that existing among sectors over time (i.e., higher F_CT_ in the past than at present). To make data comparable across time periods, sequences for 2010 specimens were trimmed to match the 210 bp available for the 1956 specimens. As an additional way to visualize potential changes in genetic structure over time, we constructed median-joining haplotype networks for the 1956 and 2010 samples using the program PopART (http://popart.otago.ac.nz/index.shtml).

To determine whether populations of *flammigerus* and *icteronotus* have experienced demographic expansions or if these taxa have exhibited historically stable population sizes, we examined trends in effective population size through time using the Extended Bayesian Skyline Plot (EBSP) method implemented in Beast v1.7.4 [59] using sequence data for the 2010 specimens. Demographic expansions are expected if the hybrid zone originated following secondary contact and stable population sizes are expected if the zone originated by primary differentiation. Analyses used the HKY + Γ model, which was selected as the best fit to the data according to the Bayesian Information Criterion (BIC) in JModelTest v 2.1.3 [60]. We ran the analysis for 25,000,000 iterations of which the first 10% were discarded as burn-in; genealogies and model parameters were sampled every 10,000 iterations. For time calibration we assumed a lognormal relaxed clock and a cytochrome-*b* substitution rate of 2.08% divergence per million years [61]. We used the mean of the distribution of population size as a prior (parameter “demographic.populationMean”) calculated from a “Coalescent: constant time” tree prior, run with the same parameters as above. Because this analysis assumes no genetic structure within the sample, we only considered populations located between Cali and Buenaventura (i.e., from the hybrid-zone transect). We conducted an analysis in which we included data for all specimens obtained across the transect and also separate analyses for *flammigerus*-like and *icteronotus-*like specimens. As with niche modelling analyses, intermediate individuals were not included in analyses conducted separately by taxon. Skyline plots were built in R [62] with code written by Valderrama *et al.* [63].

### Phenotypic characterization

To characterize the hybrid zone phenotypically, we measured six morphological characters on museum specimens (*n* = 139 males, 83 females) with dial calipers to the nearest 0.1 mm: wing length (chord of unflattened wing from bend of wing to longest primary), exposed culmen, bill depth (at the base), bill width (at the base), tail length (from point of insertion of central rectrices to tip of longest rectrix), and tarsus length (from the joint of tarsometatarsus and tibiotarsus to the lateral edge of last undivided scute). To describe morphological variation, we reduced variation in these characters using a principal components analysis (PCA).

We characterized plumage coloration based on reflectance spectra from 400 to 700 nm measured on the rump of adult museum specimens (*n* = 144 males, 70 females) using an Ocean Optics USB4F00243 Spectrometer with the SpectraSuite software (Ocean Optics). Three color measurements were estimated for each reflectance spectrum based on segment classification analysis [64]: brightness, an index of how much light is reflected from the sample relative to a white standard; chroma, the saturation of color; and hue, which relates to the wavelength of maximum slope. These measurements were calculated using R code written by Parra [65].

### Phenotypic clines and temporal dynamics

To compare patterns of morphometric and plumage color variation among adult specimens collected at different times in a geographical context, we defined three periods based on temporal sampling gaps: prior to 1911, 1956-1986, and 2010. Hybrid zones are often studied using cline-fitting algorithms that employ Bayesian or maximum-likelihood methods to estimate parameters like cline center and width (e.g. HZAR [66], Analyse [67], ClineFit [68]), and are based on population genetic models. These approaches typically require data from multiple individuals per sampling site to properly characterize variation within and among localities; accordingly, individuals need to be sampled at (or assigned to) a set of discrete sites. This approach is possible when large numbers of specimens are available and when sampling schemes have been explicitly designed with the goal of characterizing variation in space. In our case, many historical specimens were not collected with the specific purpose of describing the *Ramphocelus* hybrid zone and were not sampled at the same set of sites across different time periods. Instead, specimens were collected largely opportunistically at multiple localties and were widely scattered across the study region, with sampling varying spatially and in terms of number of individuals over time. Therefore, because our data did not readily allow us to assign individuals to discrete sampling sites, we did not employ cline-fitting methods; instead, we described the variation in morphometrics and plumage reflectance (i.e., chroma) across the hybrid zone in different time periods using log-logistic models, commonly known as Hill functions [69]. These functions are based on a sigmoidal dose-response (variable slope) model, which describes a response variable y (i.e., character value in our study) as a function of an independent variable x (i.e., distance from the initial point of the hybrid zone) based on a four-parameter logistic equation:

$$ Y=C+\frac{\left(D+C\right)}{1+\exp \left(B\left(\log (X)-\log (E)\right)\right)} $$

Here, *D* and *C* are the Y values at the plateau’s extremes (i.e. character values at each end of the hybrid zone in our case), *B* is a coefficient denoting the steepness of the curve, and *E* corresponds to the distance along the *X* axis where 50% of the value in *Y* is observed (also denoted ED50; [69]). We used the ED50 value as an estimate of cline center, and estimated cline width as the difference between the ED10 and ED90 values. We used this criterion to estimate cline width because ED10 and ED90 define the values of phenotypes in the *X* axis beyond which there are no intermediate individuals in morphology and color. For example, only individuals with scarlet rump would be observed in distances in *X* above ED90, whereas only yellow-rumped individuals would be observed in distances below ED10. We calculated the above parameters using the *drm* and *ll.4* functions implemented in the drc package for R [70].

Due to differences in sampling effort over the hybrid zone across time periods, we evaluated the sensitivity of our estimates of cline center and width to sampling effects and calculated confidence limits for these parameters based on a bootstrapping procedure. For each time period and for both morphology and plumage reflectance data, we generated a bootstrap distribution of cline center and width estimated from 1000 data sets constructed by keeping sample size constant while resampling individuals with replacement. Because in some cases sample size was small, bootstrapping resulted in some samples in which variation was not clinal or in which estimates of cline parameters were unrealistic given the geographic extent of the hybrid zone; therefore, we only retained bootstrap samples in which the estimated cline center took values between 0 and 140 km. We compared estimates of cline center and width across the tree time periods using analyses of variance (ANOVA) and post-hoc Tukey tests treating estimates obtained in bootstrap samples as replicates. Likely due to a low number of individuals with morphological data for the western extreme of the hybrid zone in 2010, bootstrap estimates of cline width for this time period were highly variable and unrealistic; therefore, we do not report confidence limits for this parameter and did not include it in the ANOVA.

### Validation of Hill-function methods

To validate the use of Hill functions to describe phenotypic and genetic clines and to infer cline center and width parameters, we compared our estimates based on Hill functions to estimates obtained by widely used cline-fitting algorithms (HZAR and Analyze) on a published data set that has been subject to cline analyses, namely the molecular data of seven loci of the *Manacus* hybrid zone in Panama [41]. We used the published allele frequency data [41] to estimate cline centers and widths using our approach and compared such estimates to published parameters [66]. Consistent estimates of parameters across methods would validate our Hill-function approach as an alternative method to estimate hybrid zone cline in scenarios where traditional algorithms cannot be used.

## Results

### Biogeographic history

A potential distribution model developed under current climatic conditions in Maxent accurately predicted the present-day distributions of *flammigerus* + *icteronotus,* with an area under the ROC-curve score of 0.987. Because this suggests that the assumption that climate limits distributions in these taxa is reasonable, we projected models onto past climatic conditions to estimate the potential historical distributions of *flammigerus* + *icteronotus* as well as those of each taxon separately at 6000 and 21,000 years ago.

**Fig. 3:**
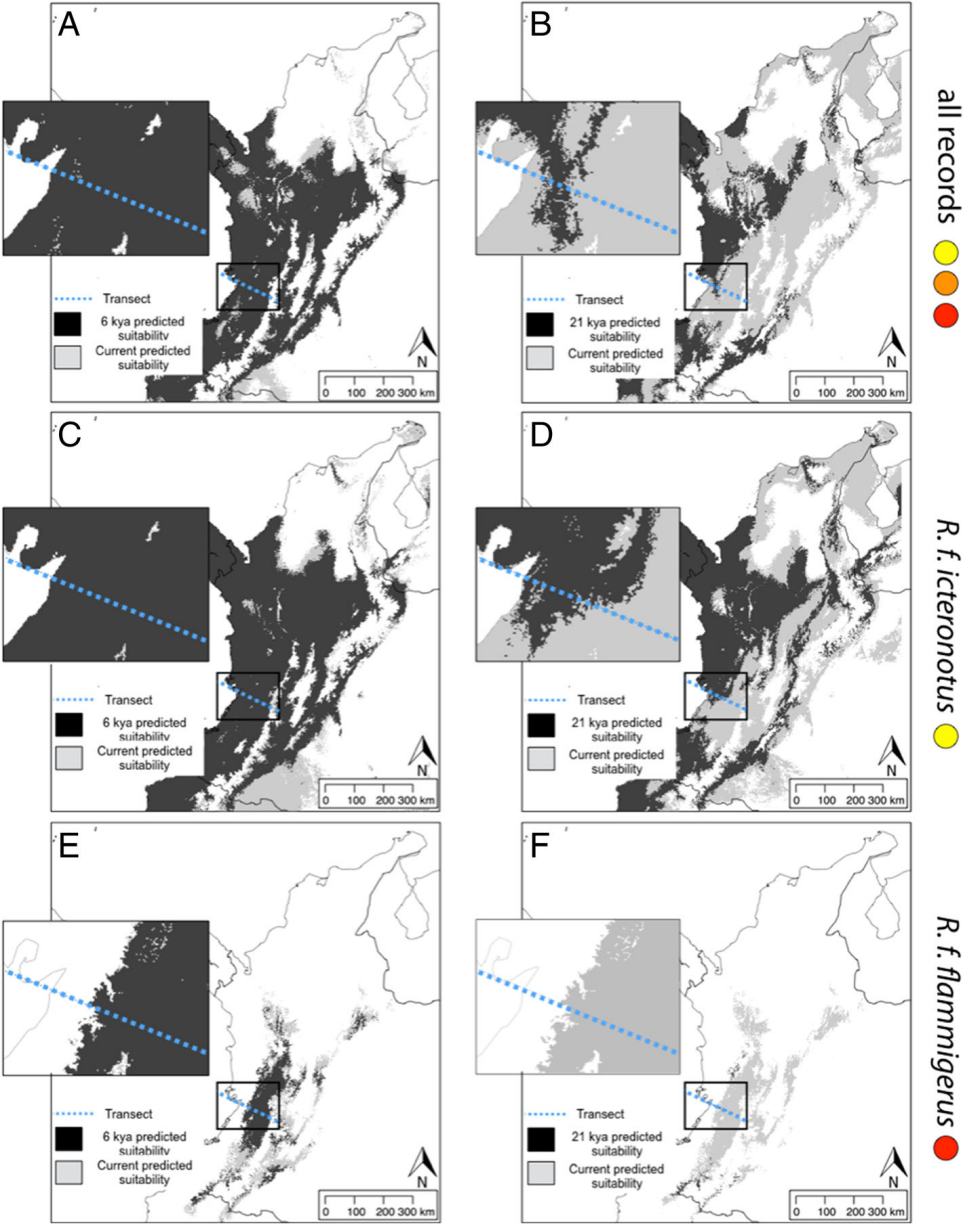
Potential distributions of *R. flammigerus* predicted by MaxEnt using climatic data. Dark gray areas show suitable climatic conditions for the occurrence of *flammigerus* and *icteronotus* (**a**) 6000 years ago and (**b**) 21,000 years ago (LGM) as estimated employing niche models including all locality data. Light gray depicts climatically suitable areas for their occurrence at present. Note the smaller predicted range during the LGM and that the two forms likely did not exhibit a continuous range along the transect (dotted line) at that time, relative to the more extensive and continuous range modeled for 6000 y.a. and under current conditions. Potential distributions are also shown separately by taxon, as predicted by models excluding intermediate specimens and considering only localities of *icteronotus* (**c** and **d**) and *flammigerus* (**e** and **f**). Note that for both taxa, distributions likely spanned the hybrid-zone transect 6000 years ago but not 21,000 years ago; in the LGM, the potential distribution of *flammigerus* (**f**) was dramatically reduced

Although our models evidently overpredict potential distributions, it is clear that the extent of suitable environments for *flammigerus* + *icteronotus* has not been stable over time. The modeled potential range of these taxa combined at present in the Northern Andes Ecoregion extends for c. 420,000 km^2^. The predicted potential distribution based on climate for 6000 years ago was of similar size, with c. 460,000 km^2^ (Fig. 3a). This indicates that current climatic conditions and those from 6000 years ago were similarly suitable for the presence of these taxa across the study region. Indeed, models suggest that the two forms could have been in contact at that time in the current location of the hybrid zone (Fig. 3a). In contrast, the predicted range during the LGM was considerably smaller than the predicted current range (c. 280,000 km^2^; Fig. 3b). Moreover, suitable conditions for *flammigerus* + *icteronotus* 21,000 years before present were not continuous along the Cordillera Occidental, suggesting that these two forms were likely disjunct during the LGM. Analyzing data separately by taxon further revealed that effects of climate on potential distributions likely differed between taxa and regions. The modelled potential distribution of *icteronotus* during the LGM was relatively extensive and covered much of the region where the hybrid zone is presently located; however, it did not fully extend to cover the southwest section of our study transect (Fig. 3d). In turn, the modelled potential distribution of *flammigerus* during the LGM was dramatically reduced relative to its potential distribution at present, with suitable environments for its occurrence at the LGM being found only in small areas in regions to the southeast and north of the present location of the hybrid zone and not at all along our transect (Fig. 3d). Taken together, niche models thus suggest that the hybrid zone may have originated via secondary contact as a result of climatic change since the LGM causing population expansions, particularly of *flammigerus* from the Cauca Valley. We address the likelihood of this historical scenario below based on patterns of genetic variation.

### Genetic characterization

**Fig. 4:**
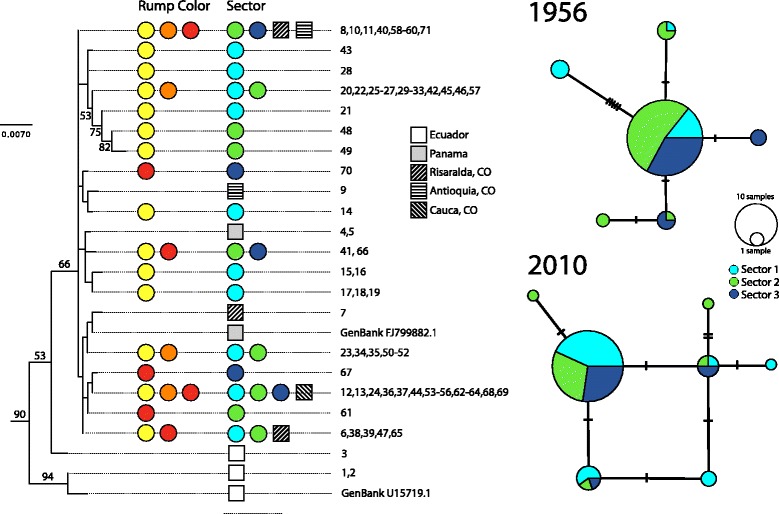
Genealogical relationships of specimens of *R. flammigerus* showing limited geographic structuring and relatively low levels of sequence divergence among haplotypes. The phylogenetic tree on the left depicts relationships among nearly complete sequences of the cytochrome *b* gene obtained for individuals from the hybrid zone and other localitites inferred using maximum-likelihood (outgroups not shown); bootstrap values on nodes are shown when ≥ 50%. Colored circles indicate a qualitative assessment of the rump color (yellow, orange and red as in individuals 1-6, 7-10 and 11-14 in Fig. 1, respectively) and location in the hybrid zone (cyan, sector 1; green, sector 2; dark blue, sector 3) of individuals from the study transect exhibiting each haplotype. The numbers correspond to specimen identifications in Additional file 1: Table S1; all numbers refer to specimens from the hybrid-zone transect unless otherwise noted. Localities outside the transect in different provinces of Colombia (CO), Ecuador, and Panama are indicated with squares. Haplotype networks on the right focused on specimens from the hybrid zone show that genetic variation in 210 bp of the cytochrome *b* gene was not clearly consistent with position of individuals along the hybrid zone in 1956 (top) or in the present (bottom), although analyses of molecular variance suggest differences in patterns of genetic structure across time periods (see text); circle sizes are proportional to the number of individuals sharing each haplotype

Overall, there was low genetic divergence between samples and genetic structure across the hybrid zone and among other localities was limited. Based on the recent samples for which we obtained long cytochrome *b* sequences, uncorrected mean sequence divergence within Colombia was only 0.3% (0-1.1%); samples from Ecuador and Colombia were 1.6% divergent, and samples from Panama and Colombia differed by only 0.4% on average. Except for a separation between samples from Ecuador and Colombia, relationships among haplotypes were not clearly resolved by the ML phylogenetic analysis, in which most nodes lacked bootstrap support and no clades associated with specific geographic regions or with plumage coloration were identified (Fig. 4). Among the long sequences (989 bp), there were 18 haplotypes with a total of 15 segregating sites in populations along our hybrid-zone transect. Among the 87 individuals from 1956 analyzed (210 bp), there were six haplotypes, with a total of nine segregating sites; uncorrected mean sequence divergence was only 0.4% (0-3.7%) and most (69) individuals shared a common haplotype. Relationships among haplotypes were not consistent with position along the hybrid zone (Fig. 4). For the same 210-bp region, there were seven haplotypes with six segregating sites in the 2010 specimens; clear structure with respect to position along the transect was not observed in the haplotype network (Fig. 4).

AMOVAs suggested that patterns of genetic structure across our study transect differ between specimens from 1956 and 2010, with greater genetic structure among sectors in the 1956 data (Table 1). For the historical data, F_CT_ values were significant (F_CT_ = 0.198, *P* = 0.011), indicating that a significant fraction of genetic variation (19%) was apportioned among sectors of the study transect. This was not the case for the present-day data, in which no genetic structure across the transect was detected (F_CT_ = 0.213, *P* = 0.111).

**Table 1:** Population genetic structure in historical and recent specimens

	1956	2010
FST		
Among localities among sectors	0.26220	0.19669
*P*-value	0.08504	0.15445
Variance components	0.34011	0.26911
% variation	73.78	80.33
FSC		
Among populations within sectors	0.07916	−0.02096
P-value	0.05181	0.24047
Variance components	0.02924	−0.00552
% variation	6.34	−1.65
FCT		
Among sectors	0.19878	0.21318
P-value	0.01173	0.11144
Variance components	0.09163	0.07141
% variation	19.88	21.32

Results are shown for analyses of molecular variance (AMOVA) based on DNA sequences of the cytochrome *b* mitochondrial gene for 87 individuals collected in 1956 and 58 individuals collected in 2010

**Fig. 5:**
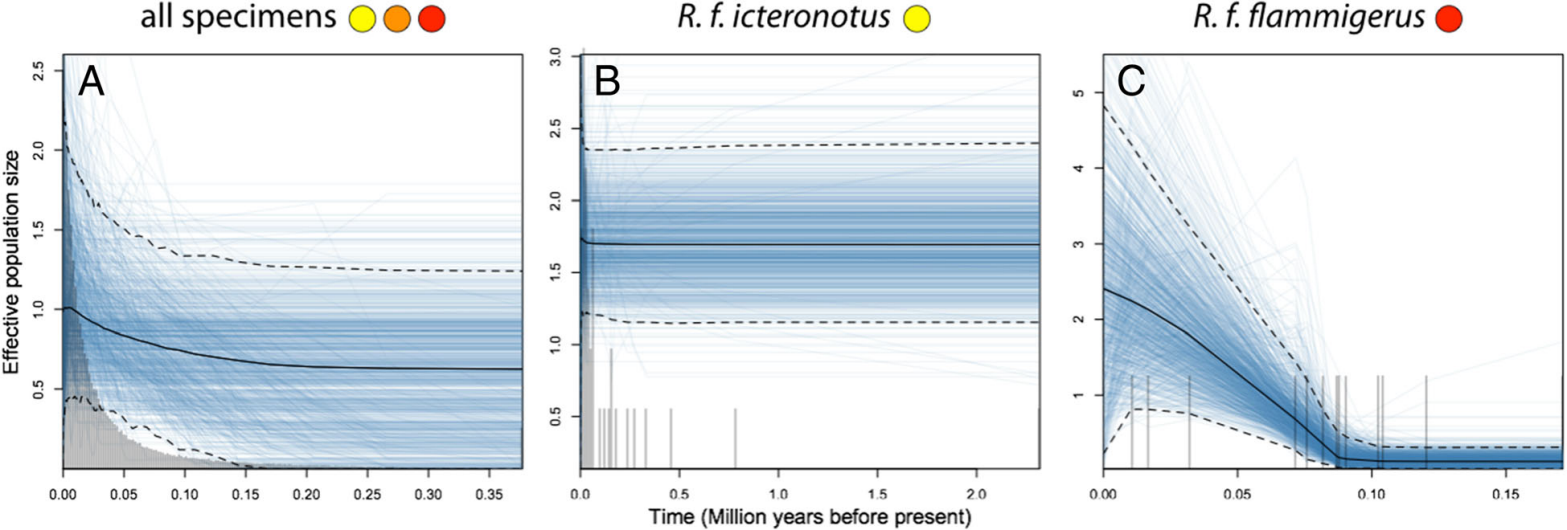
Estimates of population sizes over time obtained using the extended Bayesian skyline plot method applied to cytochrome *b* sequence data suggest demographic expansion towards the present in *R. flammigerus.* In each plot, median and credibility interval values are shown in black solid line and dashed lines, respectively. Blue lines correspond to 1000 genealogies used to estimate the 95% highest posterior density of population sizes. Bars in the histograms are proportional to the number of genealogies with values in the specific time interval. Analyses are shown for (**a**) all specimens (including *flammigerus, icteronotus* and intermediate individuals) and separately for specimens assignable to (**b**) *icteronotus* and (**c**) *flammigerus.* Although scenarios of no population change cannot be rejected for all individuals and for *icteronotus*, constant population size is firmly rejected for *flammigerus,* which shows strong evidence for demographic expansion

Although credibility intervals for population size in Bayesian skyline plots were wide, the analysis including all sequence data (“pure” and intermediate individuals combined) suggested that populations show a genetic signature of demographic expansion (Fig. 5). Constant population size cannot be firmly rejected in analyses considering all individuals due to broad credibility intervals, but because the median value of the parameter “demographic.populationSizeChanges” (PSC) differed from zero (median PSC = 1, 95% highest posterior density 0-2), the evidence points in the direction of population expansion rather than constant population size. Analyses conducted separately by taxon and excluding intermediate individuals could not reject constant population sizes in *icteronotus* (median PSC = 0, 95% highest posterior density 0-1) but strongly suggested a marked population expansion in *flammigerus* (median PSC = 1, 95% highest posterior density 1-3)*.* As with the distribution modeling analyses, these results are consistent with the hypothesis that the hybrid zone originated as a result of secondary contact following demographic expansion from formerly disjunct areas.

### Phenotypic characterization

Reduction of morphometric variation using PCA resulted in a first component (PC1) describing body size in both males and females. In both sexes, variables loading most heavily on this axis (which accounted for 24.3% of the variation in males and 34.7% in females) were tail length and wing chord. Thus, in the following we use PC1 as a general measurement of body size. We did not consider other principal component axes (e.g., PC2, on which bill dimensions loaded heavily) in additional analyses because they did not vary gradually across the hybrid zone.

Our estimates of cline parameters estimated using Hill functions were highly concordant with published estimates obtained using HZAR and Analyse in *Manacus* [66], especially for cline center (Table 2). Our estimates of cline width tended to differ more from published estimates, but in all cases the values estimated from the Hill function were within confidence intervals estimated by the other two algorithms. These results validate the use of the Hill function method to estimate cline parameters as described below.

**Table 2:**
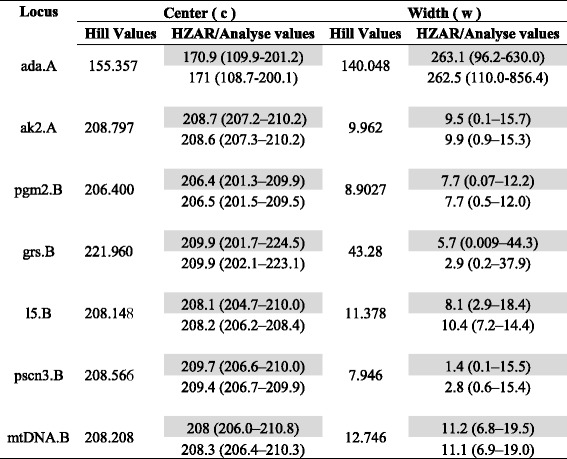
Validation of Hill-function methods as tools to estimate cline parameters

For each of seven genetic loci sampled across a *Manacus* hybrid zone in Panama, values of cline center and width estimated using Hill functions are similar to point estimates of these parameters obtained by a previous study [66] using cline-fitting algorithms HZAR (normal font) and Analyse (italic font)

**Fig. 6:**
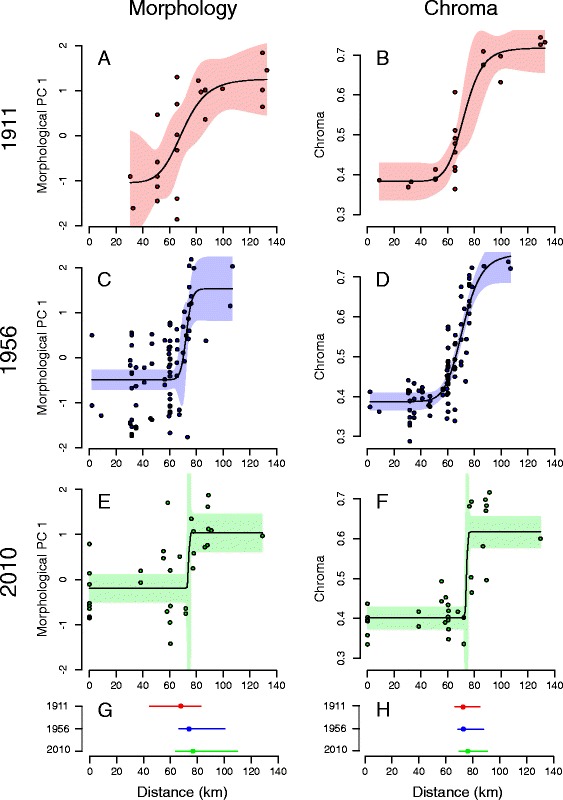
Variation in morphology and coloration of males over c. 130 km across the *Ramphocelus* hybrid zone in historical and recent specimens. **a**-**f** Circles are values for individual specimens; dark lines are clines for traits and periods in which parameters were estimated using Hill functions, with shading indicating 95% confidence intervals around cline estimates. **g**, **h** Estimates of cline centers at each time period and their confidence limits estimated using bootstrap samples, revealing coincident eastward movement of the hybrid zone over time as indicated by both traits (colors as in **a**-**f**)

**Fig. 7:**
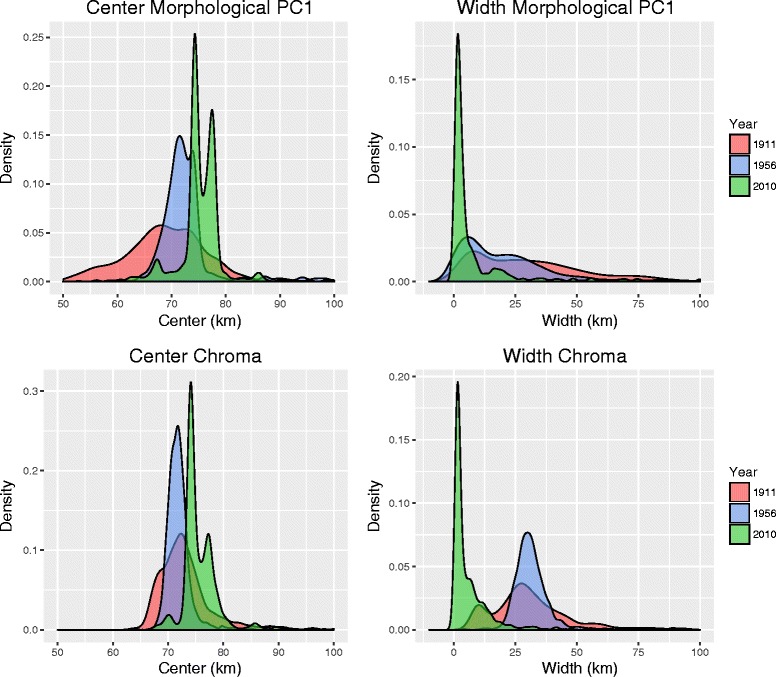
Probability density distibutions of cline center and cline width parameters estimated across 1000 bootstrap samples of specimens for the 1911 (red), 1956 (blue), and 2010 (green) time periods. Both morphological PC1 and chroma show a shift in cline center and a reduction of cline width over time

Morphological data for historical and recently collected male specimens provide evidence of clinal variation in body size (i.e. PC1) along the hybrid zone, with birds from localities to the west (*icteronotus-*type) being smaller than those from the east (*flammigerus*-type; Fig. 6). The Hill function method estimated that the center of the morphometric cline is currently located at ca. 76.7 km (mean across bootstrap replicates) from the coast extreme. The corresponding centers of the clines estimated using the pre-1911 and 1956-1986 specimens were at ca. 67.7 and 73.8 km, respectively, suggesting the zone has moved ca. 9 km over the past century (Table 3, Figs. 6 and 7, Additional file 2: Figure S1). Although differences among periods were significant (Tukey’s post-hoc test; Table 4), bootstrap estimates of uncertainty around point estimates of cline centers overlapped broadly (Figs. 6g and 7).

**Table 3:** Cline parameters estimated for morphological and coloration data in historical and recent specimens

		Center (c)	Width (w)
1911	Chroma	72.92	31.48
		(66.74-85.27)	(8.78-78.69)
	PC 1	67.67	41.75
		(44.69-82.44)	(3.61-165.4)
1956	Chroma	73.04	32.37
		(68.96-88.07)	(21.14-60.48)
	PC 1	73.85	21.97
		(66.32-100.3)	(0.84-86.43)
2010	Chroma	76.33	6.71
		(70.02-90.8)	0.75-34.07
	PC 1	76.69	33.12
		(63.95-109.84)	–

The table shows mean values of cline centers and widths obtained from bootstrap samples of morphological variation (PC1) and plumage chroma for each time period based on data for male specimens. Values in parentheses correspond to the 95% confidence intervals. All values are given in kilometers, with cline centers measured as the distance along the transect from the Pacific coast extreme (Fig. 2). No confidence limit is given for the width parameter in morphology in 2010 because bootstrapping produced unrealistic parameters (see text)

**Table 4:** Results of post-hoc ANOVA comparing of clines center (c) and widths (w) among three time periods for morphological (PC1) and plumage coloration (chroma) data using the bootstrap distributions of parameters infered from Hill functions

	Chroma				PC 1		
	Center (c)						
Year	1911	1956	2010	Year	1911	1956	2010
1911	–	0.92	**0.00**	1911	–	**0.00**	**0.00**
1956		–	**0.00**	1956		–	**0.00**
2010			–	2010			–
	Width (w)						
Year	1911	1956	2010	Year	1911	1956	2010
1911	–	0.30	**0.00**	1911	–	**0.01**	0.39
1956		–	**0.00**	1956		–	0.24
2010			–	2010			–

Numbers correspond to *P* values of Tukey’s test, with those in bold indicating significant differences between periods

Patterns of variation in color in space and time were similar to those observed for morphology. Of the three measurements of plumage coloration, chroma showed the clearest clinal pattern of variation, ranging from the yellow *icteronotus* to the redder *flammigerus* (Fig. 6). The Hill-function estimates of cline centers did not differ significantly between the pre-1911 (72.9 km) and 1956-1986 (73.0 km) periods, but the cline-center estimate for 2010 (76.3 km) was significantly different, suggesting a displacement of around 3 km in eastward direction (Tables 3 and 4, Figs. 6h and 7, Additional file 2: Figure S1).

Estimates of cline width were substantially more uncertain, but also appear to differ between the present and past. Cline width has declined across time, being wider in the pre-1911 (width 41.7 km) than in the 1956-1986 (22.0 km) and 2010 (33.1 km) specimens (Table 3, Fig. 7). Likewise, the estimated cline width in chroma at present was c. 6.7 km, but was wider in the past: 31.5 km in the pre-1911 specimens and 32.4 km 1956-1986 specimens (Table 3, Fig. 7).

Because sample sizes for females were much lower than those of males and because variation among female specimens across the hybrid zone was not clearly clinal (e.g., Additional file 3: Figure S2) we did not estimate cline parameters for female morphology and plumage measurements. Likewise, because measurements of plumage hue and brightness for males and females did not show clear clinal trends (data not shown), we did not attempt to estimate cline parameters for these traits.

## Discussion

Based on patterns of genetic variation, fossil pollen data, and ecological niche modeling, several studies in the north temperate zone indicate that the origin of hybrid zones can be explained as a result of population expansions from isolated refugia during the Quaternary [12, 13, 71]. Although a similar hypothesis was proposed to account for the origin of contact zones in tropical rainforest organisms [72, 73], research on the origin of hybrid zones in the Neotropical region has been relatively limited [74, 75]. Our niche models indicate that potential distributions of *R. f. icteronotus* and *R. f. flammigerus* were likely disjunct at the LGM (21,000 ya), but were potentially in contact by 6000 ya, likely following a marked expansion of the geographic distribution of *R. f. flammigerus.* This scenario is supported by the historical demography analysis based on mtDNA sequence data, which indicates that populations have likely experienced significant range expansions, a pattern that awaits confirmation with multilocus data (see below) that should allow for lower uncertainty in estimates of population genetic parameters. Despite the broad credibility intervals around estimates of population size through time (except in the case of *flammigerus*, which shows strong evidence for expansion), taken together with results of niche modelling, the pattern observed in skyline plots is consistent with a scenario in which forms likely diverged in isolation (*icteronotus* in the Pacific lowlands and mid elevations of the Cordillera Occidental and *flammigerus* in refugial areas of the Cauca Valley) and then met as distributions expanded, presumably tracking the influence of Pleistocene climate change on vegetation [76, 77]. A recent study also suggested that historical climatic changes likely promoted changes in the geographic distributions of lowland Neotropical birds which are presently separated by the Andes [78]. Our data also suggest that the divergence between the hybridizing *Ramphocelus* populations likely occurred in the Pleistocene, as indicated by low levels of mtDNA divergence suggesting recent differentiation. However, because Quaternary climatic oscillations started well before the LGM [79], it is possible that distribution ranges became disjunct and reconnected repeatedly at various times throughout the Pleistocene.

In contrast to our proposed scenario suggesting the origin of the *Ramphocelus* hybrid zone may date to at least 6000 before present, Sibley [25] hypothesized that contact between *flammigerus* and *icteronotus* resulted from recent anthropogenic deforestation and expansion of crops creating scrub and second-growth habitats, which are favored by these tanagers over dense rain forest. Although our analyses suggest that climatic conditions were suitable for contact between these forms thousands of years prior to major human-caused alterations in the area, it is likely that anthropogenic activities have facilitated contact between them, possibly leading to an increased incidence of hybridization in recent times.

A recent study on a hybrid zone between *Heliconius* butterflies located in the same geographic region where we studied hybridization in *Ramphocelus* also provided evidence consistent with the hypothesis of origin via secondary contact [80]. Because there are additional documented cases of hybridization in the same general area of southwestern Colombia (e.g., other *Heliconius* butterfiles [81], *Oophaga* poison frogs [82]), work on the history of the region is necessary to better understand the origin and maintenance of hybrid zones across taxa [12, 15].

Our analyses are consistent with Sibley’s [25] overall characterization of the *Ramphocelus* hybrid zone: there is clinal variation in coloration and body size, with males exhibiting clearer trends than females (see also [29]). With the caveat that uncertainty around parameter estimates is broad, two main additional insights are provided by our cline analyses. First, our data consistently indicate that for each period, clines for morphology and chroma are coincident (i.e., they have equal or very similar centers). Second, variation in morphological PC1 and chroma followed the same trend over time: for both traits, clines appear to have moved slightly to the east and to have become narrower from the past to the present.

The coincidence of cline centers for different characters and their apparent concordance in width in each of the three periods is consistent with a tension-zone model. This is further supported by the width estimated for character clines. Assuming that the hybrid zone originated at least 6000 ya according to climate-based models, that generation time in *Ramphocelus* is 1-2 years, and that dispersal distances per generation lie somewhere between 1 and 20 km, the expected width of clines under a neutral diffusion model (equations in [7, 83]) would be between c. 25 km and more than 500 km. This is much wider than what we estimated (Table 2), which suggests some form of selection is acting. Alternatively, narrow clines may be a result of a more recent origin of the hybrid zone than implied by climate data. However, even if one assumes that the hybrid zone is as young as proposed by Sibley [25], the estimated clines appear narrower than expected under neutral diffusion (c. 4-75 km). In sum, our results lead us to hypothesize that the *Ramphocelus* hybrid zone is likely a tension zone, maintained by a balance between dispersal and barriers to gene flow [1]. This interpretation contrasts with Sibley’s [25] conclusions that “gene exchange appears to be unimpeded” and that there is “no evidence of selection against the hybrids”, which he reached based on clinal patterns of variation in coloration and body size, and on the high abundance of putative hybrids. Future studies should further evaluate our tension-zone hypothesis by examining survival and mating success of intermediate phenotypes resulting from hybridization relative to parental types. In addition, detailed characterizations of patterns of variation along other contact zones between *flammigerus* and *icteronotus* located north of our study region would be of interest to determine whether intrinsic barriers to gene exchange between these taxa may exist independently of geographic or ecological context [84].

That cline centers do not appear to be coincident across different periods for each of the characters suggests movement of the *Ramphocelus* hybrid zone, but this interpretation needs to be tempered because samples were not taken at exactly the same localities at each time period and because the centers we estimated in some cases had wide support limits. In addition, our results should be interpreted with care given that the inferred geographic displacement of cline centers has been slight, corresponding to only c. 3 km between consecutive time periods. Nonetheless, our field observations indicate that the current patterns of variation are, in fact, different from those described by Sibley [25]. Specifically, we frequently observed yellow-rumped individuals at Salado and Queremal, where Sibley did not report any, and we also have occasional records of yellow-rumped individuals near Cali, the eastern extreme of the transect. Thus, our quantitative analyses and field observations suggest that the *icteronotus* phenotype (yellow rump and smaller body size) has indeed extended to the east. Several previous studies have also reported on moving tension zones [85–87] although others documented spatial stability [88, 89].

A possible explanation for the concurrent movement of clines for different characters in hybrid zones is competitive advantage of one phenotype mediated by aggression or by sexual selection [18]. Thus, movement of the *Ramphocelus* hybrid zone may have been driven by competition or sexual selection favoring the *icteronotus* phenotype. We believe it is unlikely that aggressive superiority of *icteronotus* explains this pattern as shown in other studies of hybrid zones owing to its smaller body size, although we note that in a *Manacus* (Pipridae) hybrid zone in Panama, the smaller *M. vitellinus* is more aggressive than the larger *M. candei* [90]. Thus, it would be of interest to study mating patterns in the field and to conduct mate-choice experiments to determine whether the *icteronotus* phenotype has increased reproductive success as a result of sexual selection via male-male dominance or female choice [91, 92].

If sexual selection is based on carotenoid plumage color, which presumably is strongly influenced by the environment [33], then it is somewhat puzzling that coloration seems to have moved across the hybrid zone in concert with morphometric variation, which presumably has high heritability and is not known to be involved in mate choice. It is possible, however, that the ability to obtain, accumulate and metabolize carotenoids has a heritable genetic basis [93] and that the fitness advantages it confers may be linked to genes involved in reproductive isolation [94]. If this were the case in *R. flammigerus*, then it would represent a plausible explanation for the likely movement of coloration in concert with others traits.

An alternative explanation for zone movement was proposed by Sibley [25], who predicted that the *icteronotus* phenotype would introgress across the hybrid zone as a consequence of increased gene flow from coastal to interior populations resulting from larger populations sizes in the former. This could be studied in the future with multilocus estimates of effective population sizes and of the magnitude of gene flow in both directions [95]. In addition, if deforestation has indeed resulted in increases in population size as hypothesized by Sibley [25], then movement of the hybrid zone may also partly reflect anthropogenic influences. Because hybrid zones tend to become entrapped in areas of low population densities acting as sinks for migration [1, 89], any changes in population size related to habitat modification may have partly facilitated the observed movement of the *Ramphocelus* hybrid zone.

We note that the inferred slight movement of the hybrid zone involves not only a west-east displacement, but also a shift in its center to higher elevations in the Andes. Because the elevational ranges of tropical birds may shift upslope in response to global warming [96], it is also possible that the movement of the hybrid zone is related to climatic change over the past few decades [18, 22, 97, 98], as recently documented for hybrid zones between woodpecker (Picidae) and chickadee (Paridae) species in North America [99, 100].

In contrast to multiple studies on hybridization in birds finding significant mtDNA divergence between populations located away from the center of hybrid zones and clinal variation in haplotype frequencies across them (e.g., [41, 45, 55, 74, 88, 101–105]), mtDNA variation was not geographically structured in our study system, a likely consequence of recent divergence of the hybridizing populations or of high levels of introgression. In addition, because the probability of attaining genetic differentiation between isolated populations (i.e., significant differences in allele frequencies or even reciprocal monophyly) is a function of time and effective population sizes [106], the lack of substantial mtDNA divergence along the *Ramphocelus* hybrid zone may have resulted from a short duration of the allopatric phase relative to effective population sizes. Because no clinal mtDNA variation was observed across the *Ramphocelus* hybrid zone, we were unable to estimate cline parameters for different time periods to examine zone movement as done in a few studies on hybrid zones examining genetic variation in specimens collected at different times [23, 87, 103, 107]. However, our analyses revealing more limited genetic structure across the zone as indicated by F-statistics in 2010 relative to 1956 provide some preliminary evidence that patterns of genetic variation may have not been stable over time. We hypothesize that these results may reflect an increase in introgression of mtDNA across our study transect since Sibley’s [25] time, but we acknowledge that because we only assayed a small fragment of mtDNA and because sampling was not spatially even between time periods, drawing any conclusion at this time would be premature. Nonetheless, our preliminary genetic data suggest that assessing temporal variation in spatial genetic structure using genome-wide markers (cf. [44]) would represent a fruitful avenue to better understand the ecological and evolutionary forces at work in this moving hybrid zone. Also, if shallow mtDNA divergence reflects overall patterns in genome-wide neutral divergence, then the *Ramphocelus* hybrid zone appears especially well-suited for analyses of variation in functionally important genes, which may provide important insights about the genetic basis of phenotypic variation. Recent work on other avian systems has revealed that phenotypic divergence in plumage traits controlled by relatively few loci may persist despite high overall genomic similarity [108–110].

Finally, our study serves to illustrate that Hill functions and bootstrap resampling are useful statistical tools to characterize patterns of variation across hybrid zones. In contrast to other studies [23], our work was not systematically designed to examine changes in the structure of the *Ramphocelus* hybrid zone over time, with repeated sampling of multiple specimens at fixed locations; rather, specimens were collected by numerous researchers, often opportunistically, for different purposes, and with no common sampling schemes at different times. Therefore, we did not feel confident in assigning individuals to discrete localities as required for cline-fitting algorithms which rely on calculating means and variances for samples of specimens from the same sites [66–68]. Our reanalysis of a previously published data set suggests that cline parameters estimated using Hill functions mirror closely those estimated by software commonly employed in studies of hybrid zones, suggesting such functions are useful alternatives when methods designed to study clines cannot be employed due to the nature of the available data. Although Hill functions have been used mainly to model dose-response curves, in principle they can be used to describe any relationship between variables that follows a sigmoid shape. In addition to not requiring data to be grouped in localities (i.e. each specimen conserves its position along sampling transects), the approach we followed does not assume normality in the data and can be run with low computational requeriments and relatively small sample sizes. On the other hand, we suspected that due to variation in the spatial distribution and number of specimens over time, inferences about temporal changes in the structure of the hybrid zone could be compromised by unequal sampling. However, by analyzing variation in bootstrap samples of specimens, we were able to account for sampling effects, finding that despite inconsistent sampling over time, there is sufficient signal in our data to document temporal changes in spatial patterns of phenotypic variation. Similar approaches could be employed to analyse temporal changes in spatial variation in genetic or phenotypic traits in other systems in which museum specimens have not been systematically collected over time and space but nonetheless contain rich historical information.

## Conclusions

Models of potential historical distributions based on climatic data and genetic signatures of demographic expansion suggested that the *Ramphocelus* hybrid zone we studied originated following secondary contact between populations that expanded their ranges out of isolated areas in the Quaternary, likely as a consequence of climatic change. Concordant patterns of variation in phenotypic characters across the hybrid zone and its narrow extent are suggestive of a tension zone, maintained by a balance between dispersal and selection against hybrids. In addition, our estimates of phenotypic cline parameters obtained using specimens collected over nearly a century revealed that the zone has likely moved to the east and to higher elevations, and has likely become narrower. These observed changes in the hybrid zone may be a result of sexual selection, asymmetric gene flow, or environmental change. Our data represent a baseline for a variety of ecological, behavioral, and genetic studies that could shed further light on the forces involved in speciation and hybrid-zone dynamics in this system.

## Additional files

Additional file 1: Table S1. Information on the samples of *Ramphocelus flammigerus* included in the study. (DOC 450 kb)

Additional file 2: Figure S1. Clines for morphological and plumage data estimated across 1000 bootstrap samples. The vertical line in each plot corresponds to the mean value of cline centers for the 1911 (A, B), 1956 (C, D) and 2010 (E, F) periods. (PDF 4613 kb)

Additional file 3: Figure S2. Variation in morphology (morphological PC1) and plumage chroma in female specimens across the *Ramphocelus flammigerus* hybrid zone in southwestern Colombia. Data are shown separately for historical specimens (A, B: 1911; C, D: 1956) and recent specimens (E, F: 2010). (PDF 538 kb)
