# Supplementary material for: Origin and cross-century dynamics of an avian hybrid zone
Source: BMC Evol Biol. 2017 Dec 15;17:257. doi: 10.1186/s12862-017-1096-7 (PMC5732383; doi:10.1186/s12862-017-1096-7)
Supplement: Supplementary file 1 — Information on the samples of Ramphocelus flammigerus included in the study. (DOC 450 kb) [file 12862_2017_1096_MOESM1_ESM.doc]

**Table S1** Information on the samples of *Ramphocelus flammigerus* included in the study, including identification number (ID), locality, museum catalogue number, geographical coordinates, and GenBank accession numbers for cytb sequences. Museum acronyms are as follows: LSUMZ: Louisiana State University Museum of Natural Science; ANDES-O: Museo de Historia Natural, Universidad de los Andes CUMV: Cornell University Museum of Vertebrates, Cornell University.

| **Id** | **Locality** | **Catalogue No.** | **Latitude** | **Longitude** | **Accession No.** |
| --- | --- | --- | --- | --- | --- |
| 1 | Ecuador, Esmeraldas Province El Placer | LSUMZ B-12014 | 0.867 | -78.550 | KR869689 |
| 2 | Ecuador, Esmeraldas Province, El Placer | LSUMZ B-12017 | 0.867 | -78.550 | KR869688 |
| 3 | Ecuador, Pichincha Province 5 km | LSUMZ B-35022 | 0.017 | -78.883 | KR869690 |
| 4 | Panamá, Colon Province: Achiote Road | LSUMZ B-28758 | 9.223 | -80.019 | KR869691 |
| 5 | Panamá, Darien Province | LSUMZ B-52947 | 8.083 | -77.883 | KR869692 |
| 6 | Colombia, Risaralda, Pueblo Rico | ANDES-O AMR106 | 5.231 | -76.084 | KR869693 |
| 7 | Colombia, Risaralda, Pueblo Rico | ANDES-O AMR107 | 5.231 | -76.084 | KR869694 |
| 8 | Colombia, Risaralda, Pueblo Rico | ANDES-O NGP93 | 5.235 | -76.085 | KR869695 |
| 9 | Colombia, Antioquia, San Roque | ANDES-O AMR118 | 6.384 | -74.992 | KR869696 |
| 10 | Colombia, Antioquia, Cocorná | ANDES-O AMR55 | 6.027 | -75.144 | KR869697 |
| 11 | Colombia, Antioquia, Cocorná | ANDES-O AMR56 | 6.027 | -75.144 | KR869698 |
| 12 | Colombia, Cauca, Popayán | ANDES-O AMR83 | 2.477 | -76.575 | KR869700 |
| 13 | Colombia, Cauca, Popayán | ANDES-O AMR84 | 2.477 | -76.575 | KR869699 |
| 14 | Colombia, Valle del Cauca, Buenaventura, La Barra | ANDES-O NGP101 | 3.963 | -77.379 | KR817417 |
| 15 | Colombia, Valle del Cauca, Buenaventura, La Barra | ANDES-O NGP99 | 3.963 | -77.379 | KR817418 |
| 16 | Colombia, Valle del Cauca, Buenaventura, La Barra | ANDES-O AFR29 | 3.963 | -77.379 | KR817419 |

| **Id** | **Locality** | **Catalogue No.** | **Latitude** | **Longitude** | **Accession No.** |
| --- | --- | --- | --- | --- | --- |
| 17 | Colombia, Valle del Cauca, Ladrilleros, Vía la Barra | ANDES-O AMR39 | 3.946 | -77.366 | KR817423 |
| 18 | Colombia, Valle del Cauca, Buenaventura, La Barra | ANDES-O AMR86 | 3.963 | -77.379 | KR817420 |
| 19 | Colombia, Valle del Cauca, Buenaventura, Ladrilleros | ANDES-O AMR40 | 3.944 | -77.366 | KR817422 |
| 20 | Colombia, Valle del Cauca, B/tura, La Barra | ANDES-O AMR112 | 3.963 | -77.379 | KR817459 |
| 21 | Colombia, Valle del Cauca, Buenaventura, La Barra | ANDES-O AMR111 | 3.959 | -77.376 | KR817428 |
| 22 | Colombia, Valle del Cauca, Buenaventura, La Barra | ANDES-O AMR87 | 3.964 | -77.379 | KR817473 |
| 23 | Colombia, Valle del Cauca, Buenaventura, Ladrilleros | ANDES-O AMR41 | 3.946 | -77.366 | KR817440 |
| 24 | Colombia, Valle del Cauca, Buenaventura | ANDES-O AMR105 | 3.848 | -77.000 | KR817442 |
| 25 | Colombia, Valle del Cauca, Buenaventura | ANDES-O AMR103 | 3.848 | -77.000 | KR817460 |
| 26 | Colombia, Valle del Cauca, Buenaventura | ANDES-O AMR104 | 3.848 | -77.000 | KR817464 |
| 27 | Colombia, Valle del Cauca, Dagua, El Placer | ANDES-O AMR95 | 3.591 | -76.814 | KR817466 |
| 28 | Colombia, Valle del Cauca, Dagua, Alto Anchicayá | ANDES-O AMR47 | 3.575 | -76.855 | KR817462 |
| 29 | Colombia, Valle del Cauca, Dagua, Alto Anchicayá | ANDES-O AMR69 | 3.573 | -76.879 | KR817472 |
| 30 | Colombia, Valle del Cauca, Dagua, Bajo | ANDES-O NGP28 | 3.609 | -76.918 | KR817463 |
| 31 | Colombia, Valle del Cauca, Dagua, Alto Anchicayá | ANDES-O AMR68 | 3.535 | -76.871 | KR817471 |
| 32 | Colombia, Valle del Cauca, Dagua, Bajo Anchicayá | ANDES-O AMR64 | 3.609 | -76.918 | KR817470 |
| 33 | Colombia, Valle del Cauca, Dagua, Alto Anchicayá | ANDES-O AMR45 | 3.574 | -76.878 | KR817474 |
| 34 | Colombia, Valle del Cauca, Dagua, Alto Anchicayá | ANDES-O NGP27 | 3.576 | -76.880 | KR817435 |
| 35 | Colombia, Valle del Cauca, Dagua, Alto Anchicayá | ANDES-O AMR66 | 3.576 | -76.880 | KR817439 |
| 36 | Colombia, Valle del Cauca, Dagua, Alto Anchicayá | ANDES-O NGP30 | 3.576 | -76.880 | KR817445 |

| **Id** | **Locality** | **Catalogue No.** | **Latitude** | **Longitude** | **Accession No.** |
| --- | --- | --- | --- | --- | --- |
| 37 | Colombia, Valle del Cauca, Dagua, Bajo Anchicayá | ANDES-O AMR65 | 3.609 | -76.918 | KR817446 |
| 38 | Colombia, Valle del Cauca, Buenaventura, Los Tubos | ANDES-O AMR42 | 3.845 | -76.793 | KR817433 |
| 39 | Colombia, Valle del Cauca, Buenaventura, La Delfina | ANDES-O AMR43 | 3.836 | -76.791 | KR817432 |
| 40 | Colombia, Valle del Cauca, Dagua, Queremal | ANDES-O AMR102 | 3.595 | -76.795 | KR817455 |
| 41 | Colombia, Valle del Cauca, Dagua, La Elsa | ANDES-O AMR98 | 3.576 | -76.756 | KR817424 |
| 42 | Colombia, Valle del Cauca, Dagua, Alto Anchicayá | ANDES-O NGP29 | 3.573 | -76.878 | KR817465 |
| 43 | Colombia, Valle del Cauca, Dagua, Alto Anchicayá | ANDES-O AMR63 | 3.576 | -76.879 | KR817430 |
| 44 | Colombia, Valle del Cauca, Dagua, La Elsa | ANDES-O AMR97 | 3.576 | -76.756 | KR817443 |
| 45 | Colombia, Valle del Cauca, Dagua, Alto Anchicayá | ANDES-O AMR48 | 3.575 | -76.879 | KR817468 |
| 46 | Colombia, Valle del Cauca, Dagua, Alto Anchicayá | ANDES-O AMR49 | 3.574 | -76.878 | KR817469 |
| 47 | Colombia, Valle del Cauca, Dagua, Alto Anchicayá | ANDES-O AMR46 | 3.575 | -76.879 | KR817431 |
| 48 | Colombia, Valle del Cauca, Dagua, Queremal | ANDES-O AMR70 | 3.528 | -76.719 | KR817421 |
| 49 | Colombia, Valle del Cauca, Dagua, Queremal | ANDES-O AMR74 | 3.527 | -76.713 | KR817426 |
| 50 | Colombia, Valle del Cauca, Dagua, Queremal | ANDES-O AMR78 | 3.526 | -76.733 | KR817436 |
| 51 | Colombia, Valle del Cauca, Dagua, Queremal | ANDES-O AMR75 | 3.526 | -76.733 | KR817437 |
| 52 | Colombia, Valle del Cauca, Dagua, Queremal | ANDES-O AMR66 | 3.576 | -76.880 | KR817439 |
| 53 | Colombia, Valle del Cauca, Dagua, Salado | ANDES-O AMR73 | 3.571 | -76.697 | KR817451 |
| 54 | Colombia, Valle del Cauca, Dagua, Queremal | ANDES-O AMR76 | 3.526 | -76.733 | KR817447 |
| 55 | Colombia, Valle del Cauca, Dagua, Queremal | ANDES-O AMR77 | 3.526 | -76.733 | KR817452 |
| 56 | Colombia, Valle del Cauca, Dagua, Queremal | ANDES-O AMR72 | 3.526 | -76.719 | KR817444 |

| **Id** | **Locality** | **Catalogue No.** | **Latitude** | **Longitude** | **Accession No.** |
| --- | --- | --- | --- | --- | --- |
| 57 | Colombia, Valle del Cauca, Cali, La Leonera | ANDES-O AMR85 | 3.455 | -76.635 | KR817467 |
| 58 | Colombia, Valle del Cauca, La Cumbre | ANDES-O AMR37 | 3.575 | -76.572 | KR817457 |
| 59 | Colombia, Valle del Cauca, La Cumbre, Chicoral | ANDES-O AMR36 | 3.566 | -76.581 | KR817458 |
| 60 | Colombia, Valle del Cauca, Dagua, San Bernardo | ANDES-O AMR52 | 3.474 | -76.647 | KR817456 |
| 61 | Colombia, Valle del Cauca, La Cumbre, Chicoral | ANDES-O AMR24 | 3.566 | -76.581 | KR817461 |
| 62 | Colombia, Valle del Cauca, La Cumbre, Chicoral | ANDES-O AMR25 | 3.566 | -76.581 | KR817453 |
| 63 | Colombia, Valle del Cauca, Cali, Peñas Blancas | ANDES-O AMR51 | 3.428 | -76.643 | KR817449 |
| 64 | Colombia, Valle del Cauca, Cali, Peñas Blancas | ANDES-O AMR50 | 3.428 | -76.643 | KR817448 |
| 65 | Colombia, Valle del Cauca, La Cumbre, Chicoral | ANDES-O AMR38 | 3.571 | -76.575 | KR817434 |
| 66 | Colombia, Valle del Cauca, Cali, Andes | ANDES-O NGP102 | 3.422 | -76.617 | KR817425 |
| 67 | Colombia, Valle del Cauca, Cali, Andes | ANDES-O AMR114 | 3.424 | -76.617 | KR817429 |
| 68 | Colombia, Valle del Cauca, Cali, La Elvira | ANDES-O AMR53 | 3.526 | -76.594 | KR817450 |
| 69 | Colombia, Valle del Cauca, Cali, Andes | ANDES-O AMR113 | 3.422 | -76.617 | KR817441 |
| 70 | Colombia, Valle del Cauca, Palmira, La Buitrera | ANDES-O AMR115 | 3.476 | -76.192 | KR817427 |
| 71 | Colombia, Valle del Cauca, Palmira, La Buitrera | ANDES-O AN006 | 3.471 | -76.189 | KR817454 |
| 72 | Colombia, Valle del Cauca, Buenaventura | CUMV 27061 | 3.794 | -76.993 | KR817483 |
| 73 | Colombia, Valle del Cauca, Buenaventura | CUMV 27062 | 3.794 | -76.993 | KR817542 |
| 74 | Colombia, Valle del Cauca, Buenaventura | CUMV 27063 | 3.794 | -76.993 | KR817543 |
| 75 | Colombia, Valle del Cauca, Buenaventura | CUMV 27064 | 3.794 | -76.993 | KR817544 |
| 76 | Colombia, Valle del Cauca, Buenaventura | CUMV 27066 | 3.844 | -77.000 | KR817476 |

| **Id** | **Locality** | **Catalogue No.** | **Latitude** | **Longitude** | **Accession No.** |
| --- | --- | --- | --- | --- | --- |
| 77 | Colombia, Valle del Cauca, Buenaventura | CUMV 27067 | 3.794 | -76.993 | KR817477 |
| 78 | Colombia, Valle del Cauca, Buenaventura, Río Anchicaya | CUMV 27037 | 3.614 | -76.905 | KR817528 |
| 79 | Colombia, Valle del Cauca, Buenaventura, Río Anchicaya | CUMV 27038 | 3.614 | -76.905 | KR817529 |
| 80 | Colombia, Valle del Cauca, Buenaventura, Río Anchicaya | CUMV 27039 | 3.614 | -76.905 | KR817475 |
| 81 | Colombia, Valle del Cauca, Buenaventura, Río Anchicaya | CUMV 27040 | 3.614 | -76.905 | KR817530 |
| 82 | Colombia, Valle del Cauca, Buenaventura, Río Anchicaya | CUMV 27041 | 3.614 | -76.905 | KR817531 |
| 83 | Colombia, Valle del Cauca, Buenaventura, Río Anchicaya | CUMV 27042 | 3.614 | -76.905 | KR817491 |
| 84 | Colombia, Valle del Cauca, Buenaventura, Zabaletas | CUMV 27069 | 3.745 | -76.953 | KR817478 |
| 85 | Colombia, Valle del Cauca, Buenaventura, Zabaletas | CUMV 27072 | 3.745 | -76.953 | KR817546 |
| 86 | Colombia, Valle del Cauca, Buenaventura, Zabaletas | CUMV 27073 | 3.745 | -76.953 | KR817547 |
| 87 | Colombia, Valle del Cauca, Buenaventura, El Placer | CUMV 27013 | 3.606 | -76.868 | KR817513 |
| 88 | Colombia, Valle del Cauca, Buenaventura, El Placer | CUMV 27015 | 3.606 | -76.868 | KR817514 |
| 89 | Colombia, Valle del Cauca, Buenaventura, El Placer | CUMV 27016 | 3.606 | -76.868 | KR817515 |
| 90 | Colombia, Valle del Cauca, Buenaventura, El Placer | CUMV 27017 | 3.606 | -76.868 | KR817479 |
| 91 | Colombia, Valle del Cauca, Buenaventura, El Placer | CUMV 27018 | 3.606 | -76.868 | KR817480 |
| 92 | Colombia, Valle del Cauca, Buenaventura, El Placer | CUMV 27020 | 3.606 | -76.868 | KR817516 |
| 93 | Colombia, Valle del Cauca, Buenaventura, El Placer | CUMV 27024 | 3.606 | -76.868 | KR817489 |
| 94 | Colombia, Valle del Cauca, Buenaventura, El Placer | CUMV 27022 | 3.606 | -76.868 | KR817518 |
| 95 | Colombia, Valle del Cauca, Buenaventura, El Placer | CUMV 27024 | 3.606 | -76.868 | KR817489 |
| 96 | Colombia, Valle del Cauca, Buenaventura, El Placer | CUMV 27026 | 3.606 | -76.868 | KR817519 |

| **Id** | **Locality** | **Catalogue No.** | **Latitude** | **Longitude** | **Accession No.** |
| --- | --- | --- | --- | --- | --- |
| 97 | Colombia, Valle del Cauca, Buenaventura, El Placer | CUMV 27027 | 3.606 | -76.868 | KR817520 |
| 98 | Colombia, Valle del Cauca, Buenaventura, El Placer | CUMV 27028 | 3.606 | -76.868 | KR817481 |
| 99 | Colombia, Valle del Cauca, Buenaventura, El Placer | CUMV 27029 | 3.606 | -76.868 | KR817495 |
| 100 | Colombia, Valle del Cauca, Buenaventura, El Placer | CUMV 27030 | 3.606 | -76.868 | KR817521 |
| 101 | Colombia, Valle del Cauca, Buenaventura, El Placer | CUMV 27031 | 3.606 | -76.868 | KR817522 |
| 102 | Colombia, Valle del Cauca, Buenaventura, El Placer | CUMV 27032 | 3.606 | -76.868 | KR817523 |
| 103 | Colombia, Valle del Cauca, Buenaventura, El Placer | CUMV 27033 | 3.606 | -76.868 | KR817524 |
| 104 | Colombia, Valle del Cauca, Buenaventura, El Placer | CUMV 27034 | 3.606 | -76.868 | KR817525 |
| 105 | Colombia, Valle del Cauca, Buenaventura, El Placer | CUMV 27035 | 3.606 | -76.868 | KR817526 |
| 106 | Colombia, Valle del Cauca, Buenaventura, El Placer | CUMV 27036 | 3.606 | -76.868 | KR817527 |
| 107 | Colombia, Valle del Cauca, Dagua, Queremal | CUMV 27044 | 3.517 | -76.717 | KR817488 |
| 108 | Colombia, Valle del Cauca, Dagua, La Elsa | CUMV 27046 | 3.583 | -76.774 | KR817494 |
| 109 | Colombia, Valle del Cauca, Buenaventura, Río Anchicaya | CUMV 27040 | 3.614 | -76.905 | KR817530 |
| 110 | Colombia, Valle del Cauca, Dagua, La Elsa | CUMV 27049 | 3.583 | -76.774 | KR817532 |
| 111 | Colombia, Valle del Cauca, Dagua, La Elsa | CUMV 27050 | 3.583 | -76.774 | KR817533 |
| 112 | Colombia, Valle del Cauca, Dagua, La Elsa | CUMV 27051 | 3.583 | -76.774 | KR817534 |
| 113 | Colombia, Valle del Cauca, Dagua, La Elsa | CUMV 27052 | 3.583 | -76.774 | KR817535 |
| 114 | Colombia, Valle del Cauca, Dagua, La Elsa | CUMV 27053 | 3.583 | -76.774 | KR817536 |
| 115 | Colombia, Valle del Cauca, Dagua, La Elsa | CUMV 27055 | 3.583 | -76.774 | KR817537 |
| 116 | Colombia, Valle del Cauca, Dagua, La Elsa | CUMV 27056 | 3.583 | -76.774 | KR817538 |

| **Id** | **Locality** | **Catalogue No.** | **Latitude** | **Longitude** | **Accession No.** |
| --- | --- | --- | --- | --- | --- |
| 117 | Colombia, Valle del Cauca, Dagua, La Elsa | CUMV 27057 | 3.583 | -76.774 | KR817539 |
| 118 | Colombia, Valle del Cauca, Dagua, La Elsa | CUMV 27058 | 3.583 | -76.774 | KR817482 |
| 119 | Colombia, Valle del Cauca, Dagua, La Elsa | CUMV 27059 | 3.583 | -76.774 | KR817540 |
| 120 | Colombia, Valle del Cauca, Dagua, La Elsa | CUMV 27060 | 3.583 | -76.774 | KR817541 |
| 121 | Colombia, Valle del Cauca, Dagua, Rio Blanco | CUMV 27068 | 3.600 | -76.817 | KR817545 |
| 122 | Colombia, Valle del Cauca, Dagua, Rio Blanco | CUMV 27074 | 3.600 | -76.817 | KR817548 |
| 123 | Colombia, Valle del Cauca, Dagua, Rio Blanco | CUMV 27076 | 3.600 | -76.817 | KR817490 |
| 124 | Colombia, Valle del Cauca, Dagua, Rio Blanco | CUMV 27078 | 3.600 | -76.817 | KR817549 |
| 125 | Colombia, Valle del Cauca, Dagua, Rio Blanco | CUMV 27079 | 3.600 | -76.817 | KR817550 |
| 126 | Colombia, Valle del Cauca, Dagua, Rio Blanco | CUMV 27082 | 3.600 | -76.817 | KR817551 |
| 127 | Colombia, Valle del Cauca, Dagua, Rio Blanco | CUMV 27083 | 3.600 | -76.817 | KR817552 |
| 128 | Colombia, Valle del Cauca, Dagua, Rio Blanco | CUMV 27084 | 3.600 | -76.817 | KR817553 |
| 129 | Colombia, Valle del Cauca, Dagua, Salado | CUMV 26992 | 3.567 | -76.717 | KR817558 |
| 130 | Colombia, Valle del Cauca, Dagua, Salado | CUMV 26993 | 3.567 | -76.717 | KR817502 |
| 131 | Colombia, Valle del Cauca, Dagua, Salado | CUMV 26994 | 3.567 | -76.717 | KR817557 |
| 132 | Colombia, Valle del Cauca, Dagua, Salado | CUMV 26995 | 3.567 | -76.717 | KR817492 |
| 133 | Colombia, Valle del Cauca, Dagua, Salado | CUMV 26996 | 3.567 | -76.717 | KR817503 |
| 134 | Colombia, Valle del Cauca, Dagua, Salado | CUMV 26997 | 3.567 | -76.717 | KR817497 |
| 135 | Colombia, Valle del Cauca, Dagua, Salado | CUMV 26998 | 3.567 | -76.717 | KR817504 |
| 136 | Colombia, Valle del Cauca, Dagua, Salado | CUMV 26999 | 3.567 | -76.717 | KR817505 |

| **Id** | **Locality** | **Catalogue No.** | **Latitude** | **Longitude** | **Accession No.** |
| --- | --- | --- | --- | --- | --- |
| 137 | Colombia, Valle del Cauca, Dagua, Salado | CUMV 27000 | 3.567 | -76.717 | KR817506 |
| 138 | Colombia, Valle del Cauca, Dagua, Salado | CUMV 27001 | 3.567 | -76.717 | KR817507 |
| 139 | Colombia, Valle del Cauca, Dagua, Salado | CUMV 27002 | 3.567 | -76.717 | KR817484 |
| 140 | Colombia, Valle del Cauca, Dagua, Salado | CUMV 27003 | 3.567 | -76.717 | KR817508 |
| 141 | Colombia, Valle del Cauca, Dagua, Salado | CUMV 27004 | 3.567 | -76.717 | KR817509 |
| 142 | Colombia, Valle del Cauca, Dagua, Salado | CUMV 27005 | 3.567 | -76.717 | KR817510 |
| 143 | Colombia, Valle del Cauca, Dagua, Salado | CUMV 27006 | 3.567 | -76.717 | KR817493 |
| 144 | Colombia, Valle del Cauca, Dagua, Salado | CUMV 27007 | 3.567 | -76.717 | KR817485 |
| 145 | Colombia, Valle del Cauca, Dagua, Salado | CUMV 27008 | 3.567 | -76.717 | KR817511 |
| 146 | Colombia, Valle del Cauca, Dagua, Salado | CUMV 27010 | 3.567 | -76.717 | KR817487 |
| 147 | Colombia, Valle del Cauca, Dagua, Salado | CUMV 27011 | 3.567 | -76.717 | KR817512 |
| 148 | Colombia, Valle del Cauca, Dagua, Salado | CUMV 27012 | 3.567 | -76.717 | KR817486 |
| 149 | Colombia, Valle del Cauca, Dagua, Queremal | CUMV 27045 | 3.517 | -76.717 | KR817496 |
| 150 | Colombia, Valle del Cauca, La Cumbre, San Antonio | CUMV 22956 | 3.500 | -76.633 | KR817498 |
| 151 | Colombia, Valle del Cauca, La Cumbre, San Antonio | CUMV 22958 | 3.500 | -76.633 | KR817499 |
| 152 | Colombia, Valle del Cauca, Palmira, La Manuelita | CUMV 22959 | 3.583 | -76.283 | KR817500 |
| 153 | Colombia, Valle del Cauca, Palmira, Miraflores | CUMV 22961 | 3.578 | -76.434 | KR817501 |
| 154 | Colombia, Valle del Cauca, Tulua, La Paila | CUMV 27086 | 4.319 | -76.072 | KR817554 |
| 155 | Colombia, Valle del Cauca, Jamundi, La Olga | CUMV 27087 | 3.299 | -76.519 | KR817555 |
| 156 | Colombia, Valle del Cauca, Buga, La Habana | CUMV 27088 | 3.881 | -76.191 | KR817556 |
