# Supplementary figures and images for: Origin and cross-century dynamics of an avian hybrid zone
Source: BMC Evol Biol. 2017 Dec 15;17:257. doi: 10.1186/s12862-017-1096-7 (PMC5732383; doi:10.1186/s12862-017-1096-7)

1911

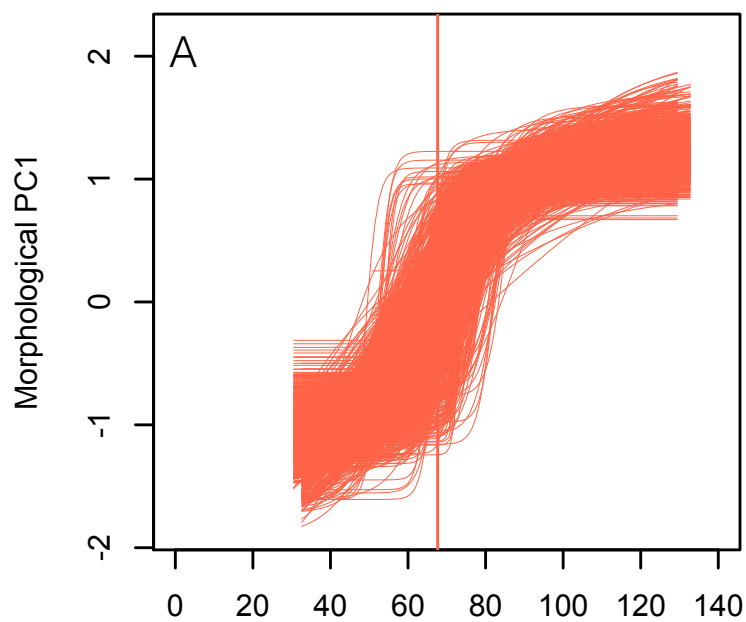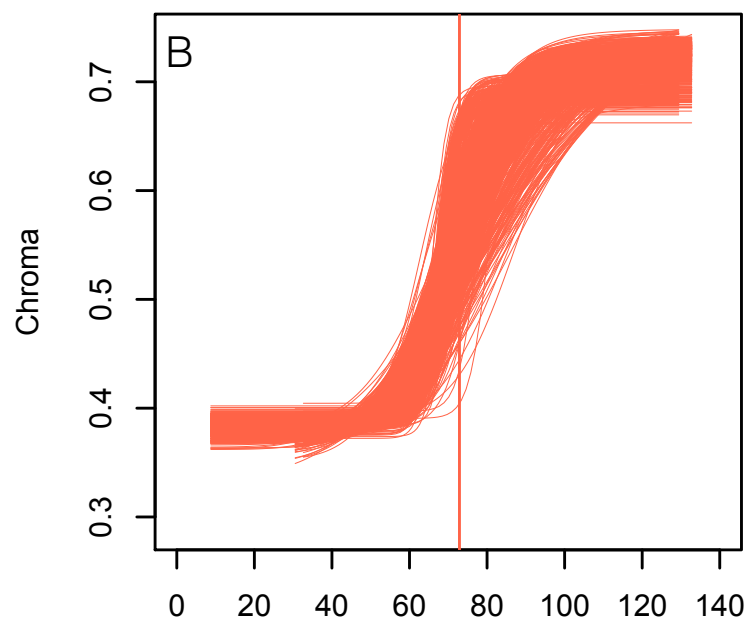

1956

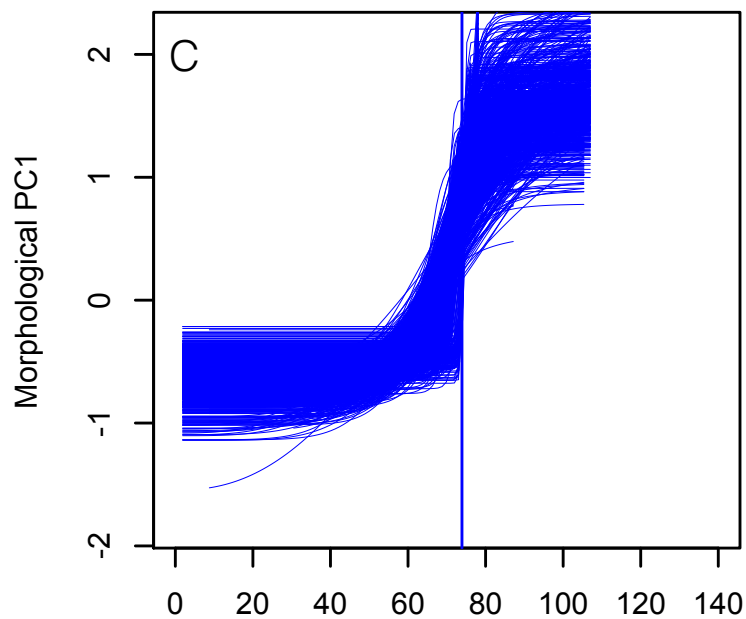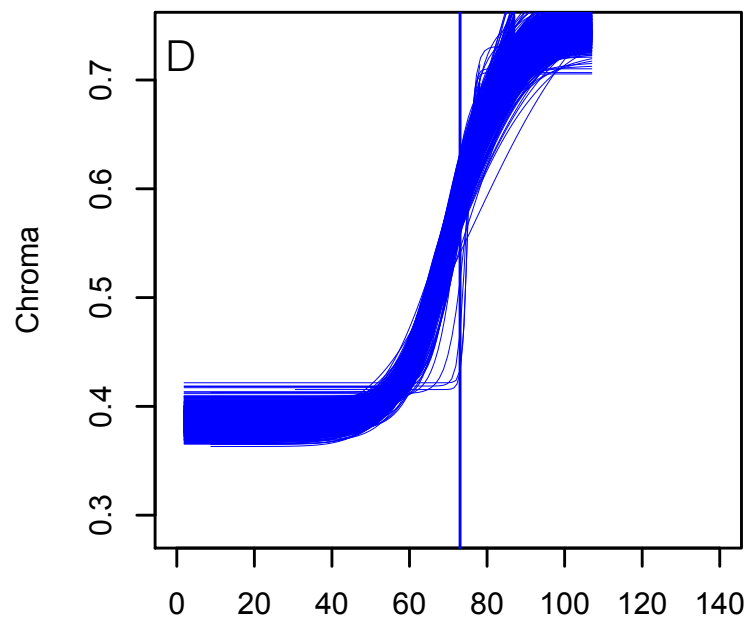

2010

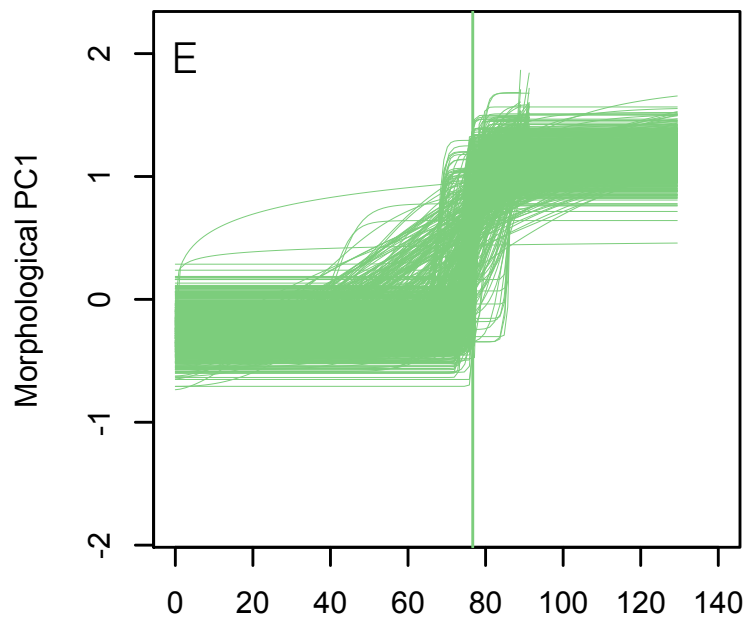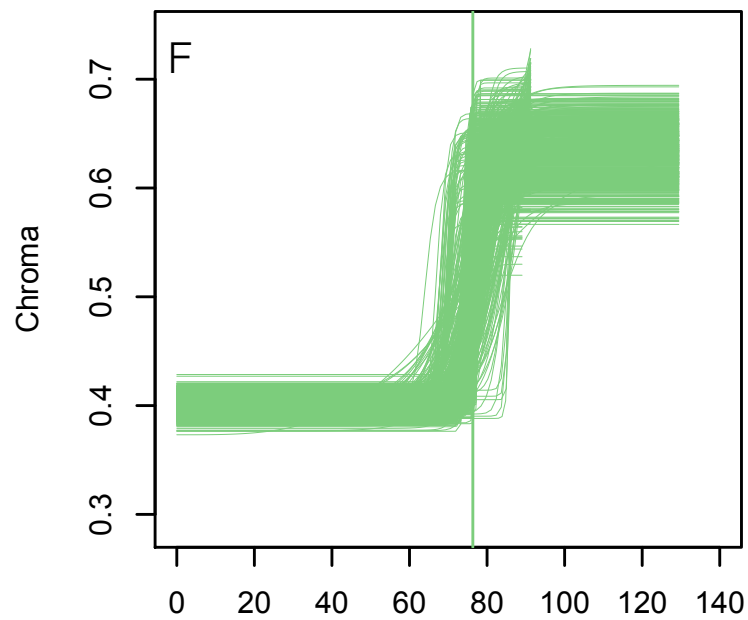

Distance (km)

Distance (km)

Supplement: Supplementary file 2 — Clines for morphological and plumage data estimated across 1000 bootstrap samples. The vertical line in each plot corresponds to the mean value of cline centers for the 1911 (A, B), 1956 (C, D) and 2010 (E, F) periods. (PDF 4613 kb) [file 12862_2017_1096_MOESM2_ESM.pdf]

## Morphology

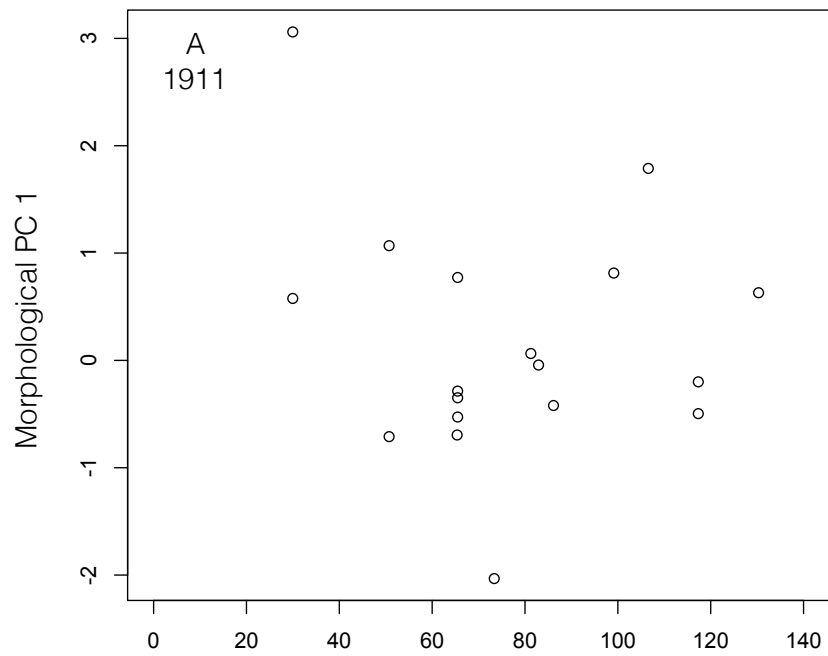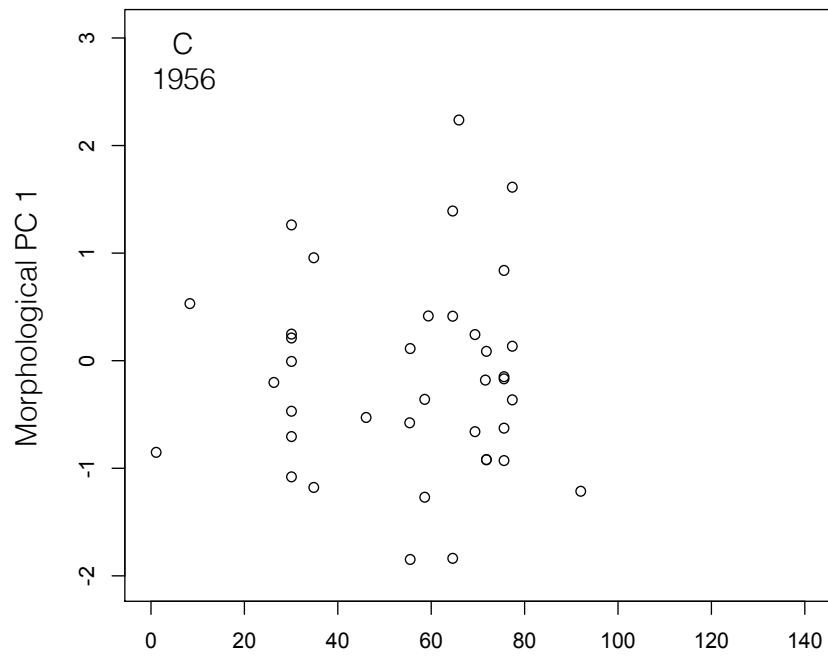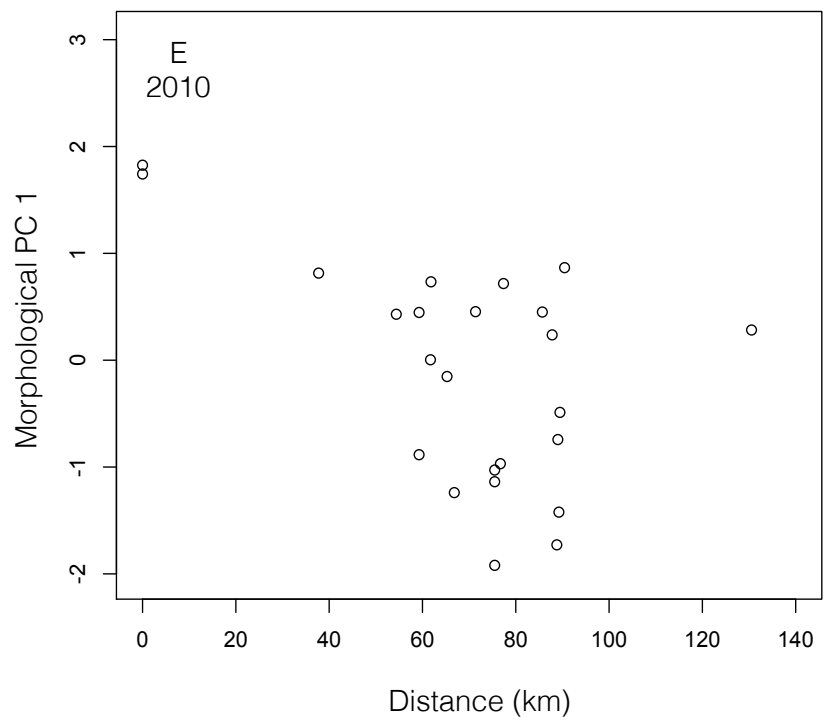

## Color

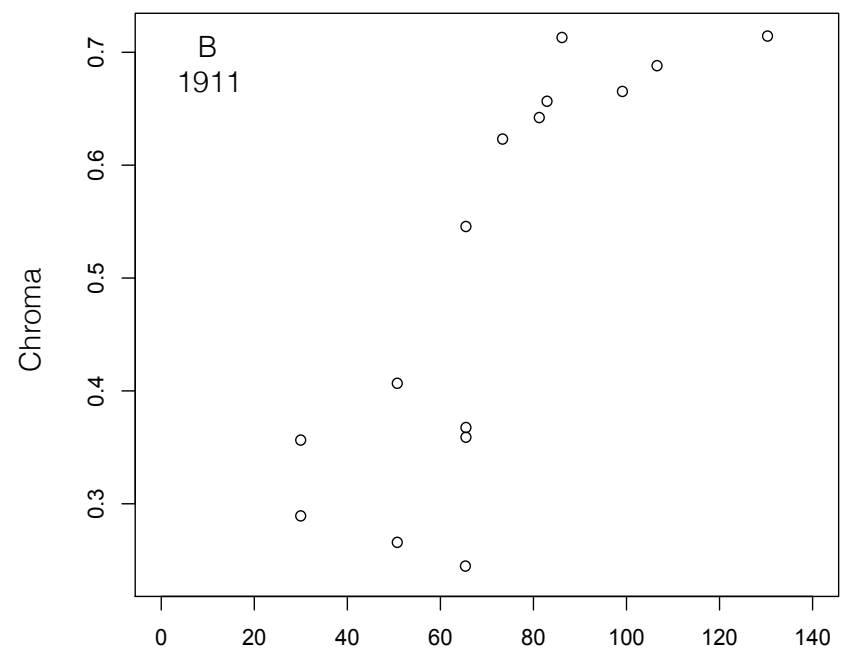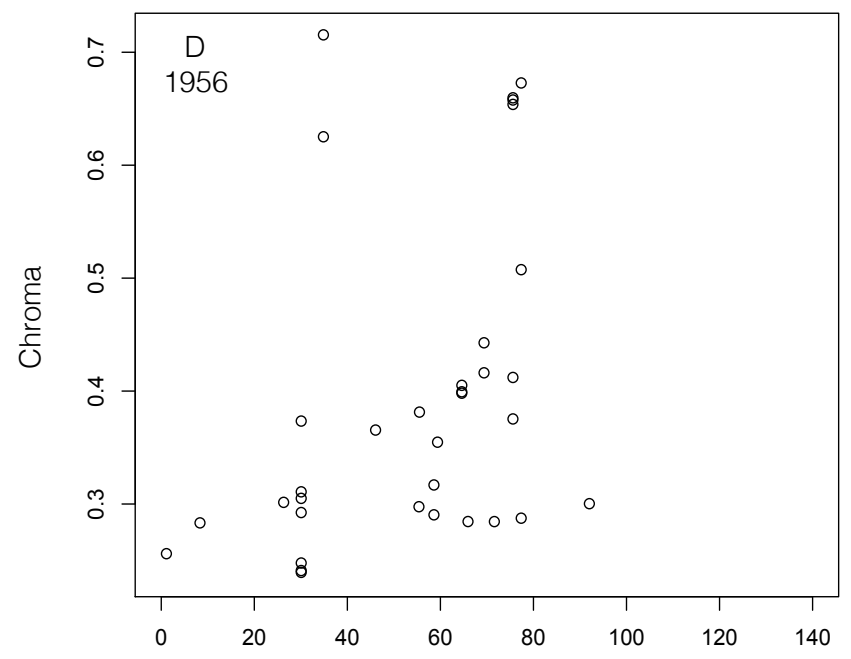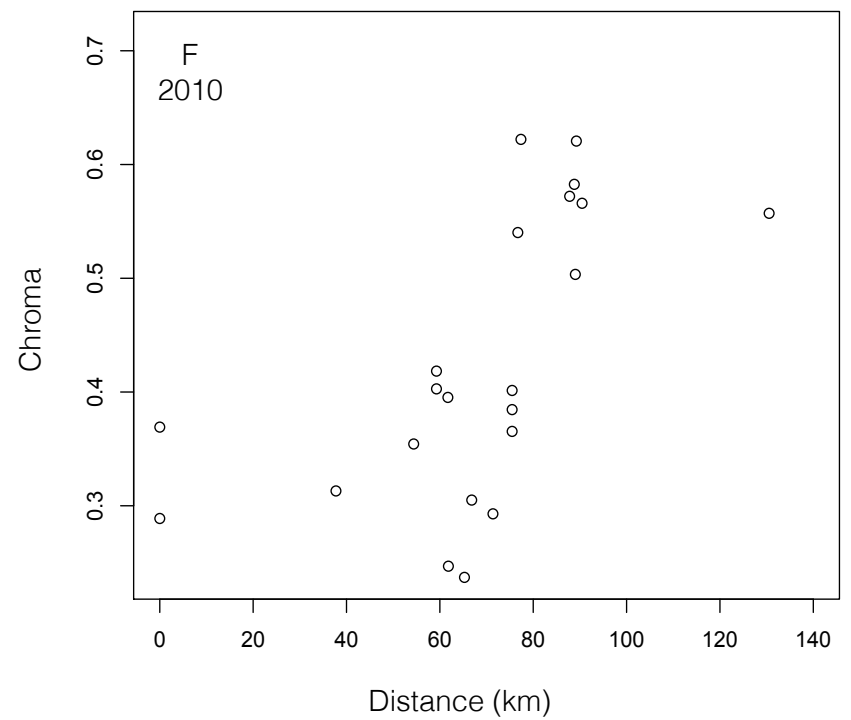

Supplement: Supplementary file 3 — Variation in morphology (morphological PC1) and plumage chroma in female specimens across the Ramphocelus flammigerus hybrid zone in southwestern Colombia. Data are shown separately for historical specimens (A, B: 1911; C, D: 1956) and recent specimens (E, F: 2010). (PDF 538 kb) [file 12862_2017_1096_MOESM3_ESM.pdf]
